# Bimodal Cholesterol for Correlative In-Cell DNP Solid-State NMR and Confocal Microscopy of the Plasma Membrane

**DOI:** 10.1021/jacs.5c20070

**Published:** 2026-04-09

**Authors:** Sarah A. Overall, Ancy T. Wilson, Dorothea Pinotsi, César Själsjö, Snorri Th Sigurdsson, Alexander B. Barnes

**Affiliations:** † Institute of Molecular Physical Science, ETH Zurich, Zurich 8093, Switzerland; ‡ Science Institute, University of Iceland, Dunhagi 3, Reykjavik 107, Iceland; § Scientific Center for Optical and Electron Microscopy, ETH Zurich, Zurich 8093, Switzerland; ∥ Swedish NMR Center, 3570 University of Gothenburg, Gothenburg 40530, Sweden

## Abstract

Cholesterol plays a pivotal role in defining the structure and signaling function of plasma membrane lipid rafts, yet direct nanoscale measurements of cholesterol-rich domains in intact cells that enable atomic scale analysis remains challenging. Here, we present AsymPol-Chol-AF647, a bimodal cholesterol-based probe that integrates a biradical (AsymPol) for Dynamic Nuclear Polarization (DNP) with a fluorescent dye (AF647), enabling correlative in-cell DNP solid-state NMR and confocal fluorescence microscopy of the plasma membrane. The cholesterol anchor ensures insertion into the plasma membrane, providing a spatially defined delivery of the polarizing agent. Confocal imaging in live cells confirms plasma membrane localization and reveals concentration-dependent perturbations to lipid raft integrity, with optimal conditions at 0.19 nmol/10^6^ cells maintaining cell viability and discrete GM1-positive microdomains (associated with lipid rafts). Under these conditions, significant global enhancements of natural abundance ^13^C of ε ≈ 14 were achieved. Analysis of signal buildup kinetics unveiled evidence for more complex hyperpolarization dynamics than the simple spherical model indicated by the microscopy. These findings lay the groundwork for future studies utilizing bimodal polarizing agents for use not just for selective cellular enhancements but also as tools for the determination of the spatial organization of cellular structures at nanometer resolution.

## Graphical abstract

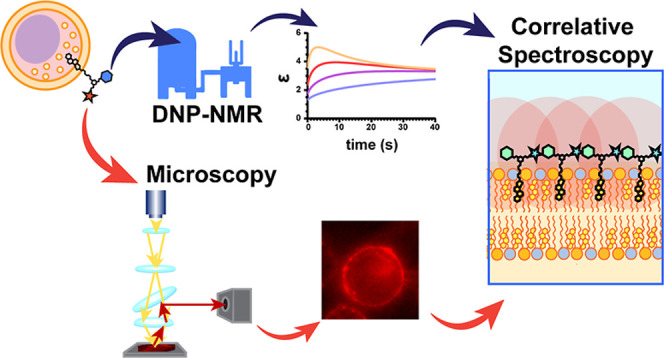

## Introduction

Cholesterol is an important component of cellular membranes, influencing membrane order parameters and membrane protein conformations.^1^ Cholesterol is also a critical component of lipid rafts, discrete liquid-ordered (Lo) microdomains within the membrane that are central to the spatiotemporal regulation of many cell signaling cascades.^2–7^ In fact, cholesterol is thought to largely determine the biophysical properties and formation of lipid rafts.^8,9^ However, the nanoscale structural properties, such as leaflet asymmetry of raft-associated cholesterol, signaling-associated domain size, and membrane-order dynamics, are largely unknown within the context of intact cells, where the composition and architecture is complex and heterogeneous. Obtaining a structural understanding of cholesterol’s role in modulation of the structure and biophysical properties of membrane microdomains within cells would provide unprecedented insight into the regulation of raft and cholesterol-associated cell signaling events. However, there are currently no techniques that can provide this kind of information on the angstrom scale besides Nuclear Magnetic Resonance (NMR) spectroscopy. Fluorescence microscopy has been an indispensable technique for understanding the dynamics of lipid rafts at the single cell level and has been particularly revealing in the complex raft patterns associated with the activation of T cells during immune responses,^7,10,11^ even down to the 50 nm scale. However, accessing angstrom scale information about cell membranes would enable a detailed structural study of cellular membranes and lipid rafts.

In-cell dynamic nuclear polarization (DNP) NMR is an emerging method for characterizing membrane-associated signaling complexes and large cellular assemblies directly within their native context.^12–14^ DNP enhances NMR sensitivity by several orders of magnitude by transferring the large polarization from unpaired electrons, usually referred to as a polarizing agent (PA), to nearby nuclei,^15,16^ enabling detection of previously inaccessible biomolecular signals. This sensitivity gain allows atomic-level structural and dynamic insights from cellular environments that have been beyond reach with conventional NMR.^17,18^ In-cell DNP-NMR offers a pathway to investigate the structural and biophysical properties of membranes and the role of small molecules, such as cholesterol, in defining these properties.^19^ A DNP-NMR-based study of lipid rafts requires radicals that specifically target lipid rafts.

A range of PAs for targeted biomolecular DNP have been developed, particularly for proteins and protein ligands, that have enabled selective enhancement of NMR signals.^20–23^ One benefit of targeted DNP for biomolecular samples is the reduction of radical aggregation upon freezing, which greatly improves DNP enhancements by extending the electron transverse relaxation time, T_2,e_. For in-cell samples, targeted DNP also offers the opportunity to reduce spectral complexity to regions and organelles of interest through site-specific enhancements, which facilitate data interpretation and analysis. Even in protein studies, selective DNP with radical-tagged ligands has demonstrated the utility of these approaches.^24^ Such PAs will be an important part of the in-cell DNP-NMR toolkit, establishing in-cell DNP-NMR as a critical method in the study of cellular membranes and their interactions with proteins within intact cells.

PAs that target specific cellular structures also offer the opportunity to investigate cellular domain sizes, geometries, and structural properties. NMR and DNP-NMR have been used to determine the domain size and distribution of amorphous materials and polymer mixtures.^25–28^ This information can be obtained by extracting the polarization buildup dynamics (with or without DNP), measured by NMR, and fitting these time-dependent distributions of spin polarization to models of spin diffusion.^26,29^ These models are built upon well-established theory of spin diffusion dynamics and have been successfully utilized in determining the domain sizes of amorphous pharmaceutical mixtures and materials under DNP conditions in which the initial spin polarization is very large due to the DNP effect.^26,29,30^ Furthermore, these models can be used to extract geometric information in regard to the heterogeneous distribution of multicomponent materials.^26,31^ Though validated down to ∼20 nm in protonated materials, a scale within which lipid rafts fall, an exploration of the application of these models and simulations for the potential extraction of cellular architectures has not been pursued. This presents an untapped avenue for probing nanoscale structures within cells simultaneously with angstrom scale investigations of membrane component interactions. Such studies require both selective doping of the cellular components of interest with PAs and the ability to extract base geometric information about the PA distribution to establish an appropriate model to simulate. However, few fluorescent-PAs for localized in-cell DNP-NMR studies have been described.^13,23^

In this paper, we describe the design and synthesis of a bimodal PA that contains a cholesterol molecule conjugated to a well-known stable biradical, AsymPol,^32^ to facilitate DNP and AF647 to enable correlative confocal microscopy and future super-resolution microscopy for determination of the spatial distribution of the biradical within cells. We demonstrate efficient DNP with AsymPol-Chol-AF647 and utilize the dynamics of hyperpolarization distribution to characterize the behavior of spin polarization within such a heterogeneous environment. We identified key parameters for monitoring localized hyperpolarization dynamics in cells that complicate even a simple core–shell model as is the case for plasma membrane localized PA, taking the first steps toward correlative microscopy and NMR spectroscopy of cellular samples.

## Results

The structure of the bimodal, cholesterol-based PA AsymPol-Chol-AF647 contains three functional elements on a tripod scaffold: (i) a nitroxide biradical PA for DNP, (ii) an alkyne-modified cholesterol for directing to membranes, and (iii) a fluorophore for localization of the PA by fluorescence microscopy (Figure 1).

**Figure 1:**
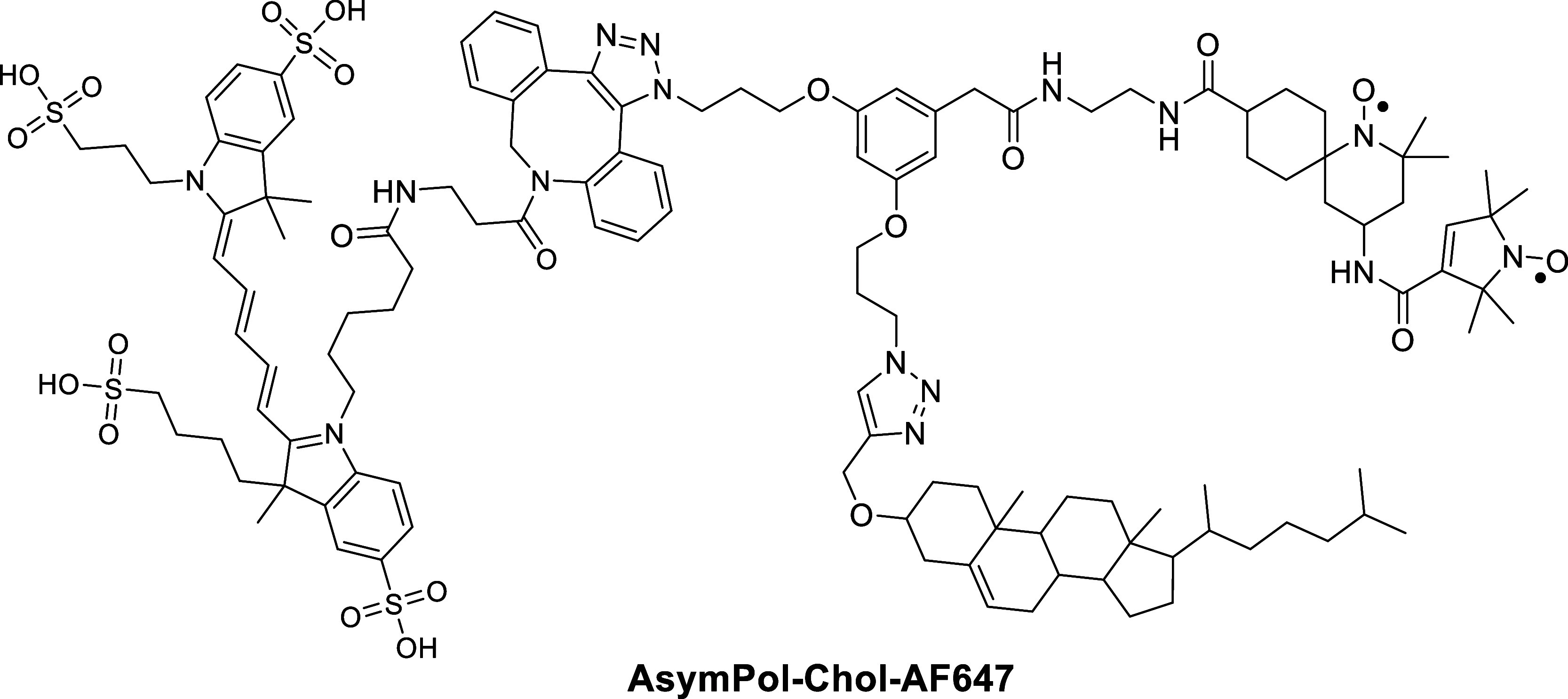
Structure of AsymPol-Chol-AF647.

### Synthesis of AsymPol-Chol-AF647

Synthesis of AsymPol-Chol-AF647 commenced with the incorporation of an azide group into **1** (Scheme 1), followed by copper­(I)-catalyzed azide–alkyne cycloaddition (CuAAC) with alkyne-modified cholesterol **3** ^33^ to afford the triazole conjugate **4**. Incorporation of a second azide moiety, followed by deprotection of the carboxylic acid under basic conditions, yielded compound **6**. Compound **6** was subsequently coupled with the amine-functionalized AsymPol biradical **7**, synthesized from the corresponding carboxylic acid^34^ with ethylene diamine (see Supporting Information-1), to afford **8**. Finally, copper-free and strain-promoted azide–alkyne cycloaddition (SPAAC) with DBCO-AF647 afforded fluorescently labeled PA AsymPol-Chol-AF647 (Scheme 1).

**Scheme 1:**
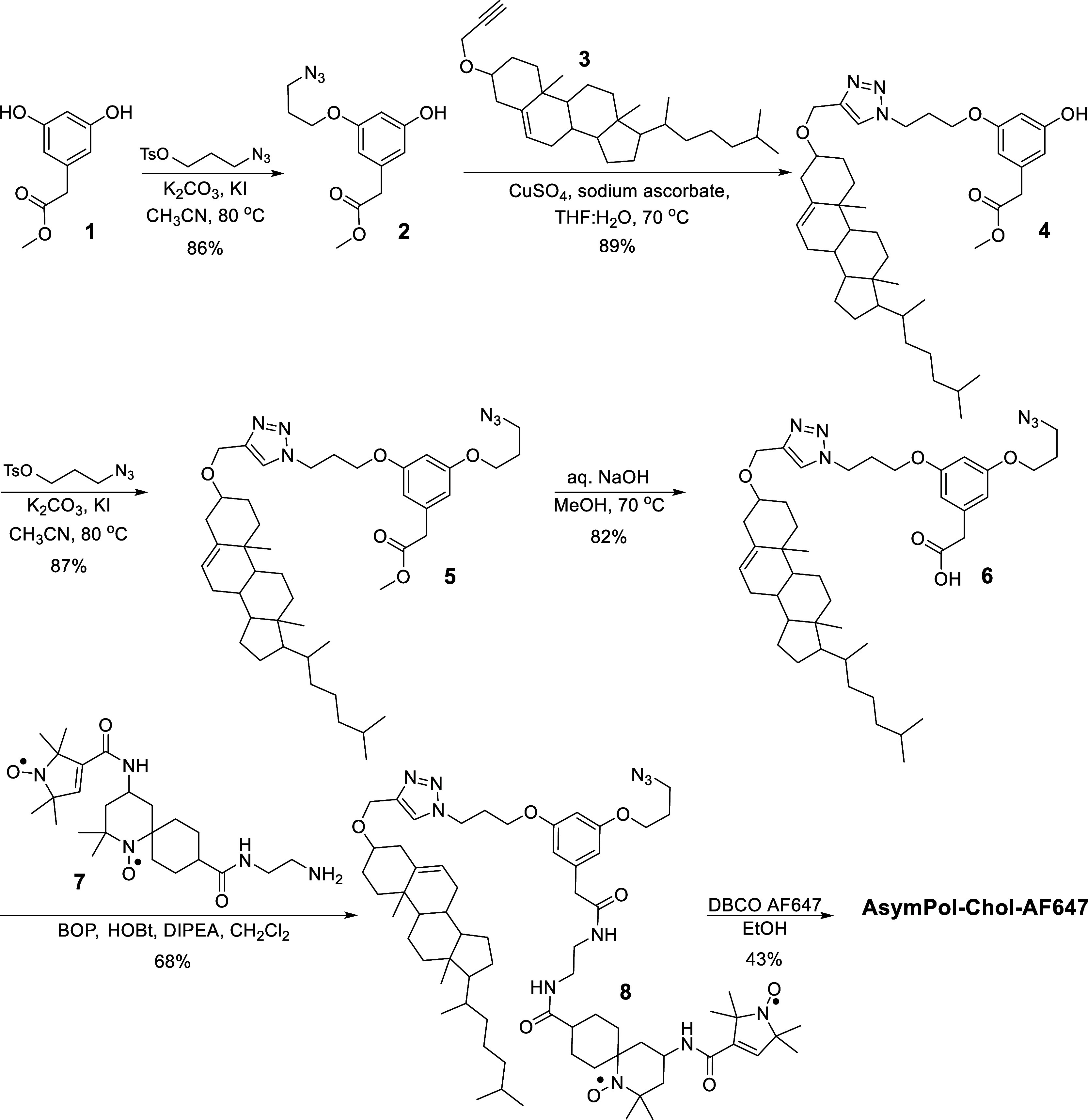
Synthesis of AsymPol-Chol-AF647

### Cellular Localization of AsymPol-Chol-AF647

To determine the cellular localization of the PA AsymPol-Chol-AF647, we incubated JLat 9.2 T cells with varying PA concentrations and performed confocal microscopy as described in the experimental methods (Supporting Information-2). At 9.67 mM (9.67 nmol/million cells), we observed immediate complete lysis of the cells. Presumably, this is due to rigidification of the plasma membrane by the insertion of large quantities of cholesterol, suggesting that the PA has a very strong affinity for membrane insertion and incorporation. This would be consistent with the hydrophobicity of this agent. Thus, radical loading of the cells will be strictly limited by the capacity of the membranes to accommodate additional cholesterol. Reducing the concentration of the PA to 480 μM (0.48 nmol/million cells) improved cell survival significantly, with the percentage of live cells increasing to 80.5% after addition of the PA, as determined by trypan blue staining (Supporting Information-3). However, this is still an increase in cell death compared with untreated cells (97.3% live cells). The confocal microscopy of the labeled cells demonstrated excellent localization of the PA to the plasma membrane (Figure 2a), with a small proportion of live cells exhibiting intracellular staining that increased with time (appearing after 20 min from the addition of the PA (Supporting Information-4)). The morphology of the intracellular staining is localized to one side of the cell in a discrete structure, most likely indicating the Golgi, which would suggest retrograde transport from the plasma membrane, consistent with the increased observation of this staining pattern over time. Dead cells exhibited uniform intracellular staining with the PA, indicative of a loss of plasma membrane semipermeability and damage, although macroscopic cell morphology was maintained (Figure 2a bright field). Co-staining with anti-GM1 antibodies (GM1 is a glycolipid strongly associated with cholesterol-rich phase separated membrane regions and is used a marker of lipid raft in cells)^35,36^ revealed some disruption to lipid raft structures at the plasma membrane of live cells (Figure 2b), evidenced by reduced GM1 staining intensity, increased uniformity of GM1 staining on the plasma membrane, and a loss of discrete GM1 positive clusters that are distinctly observed in untreated cells (Figure 2a,b). Of the GM1-positive clusters observed, these appeared to, in part, exclude the PA (Figure 2a overlay).

**Figure 2:**
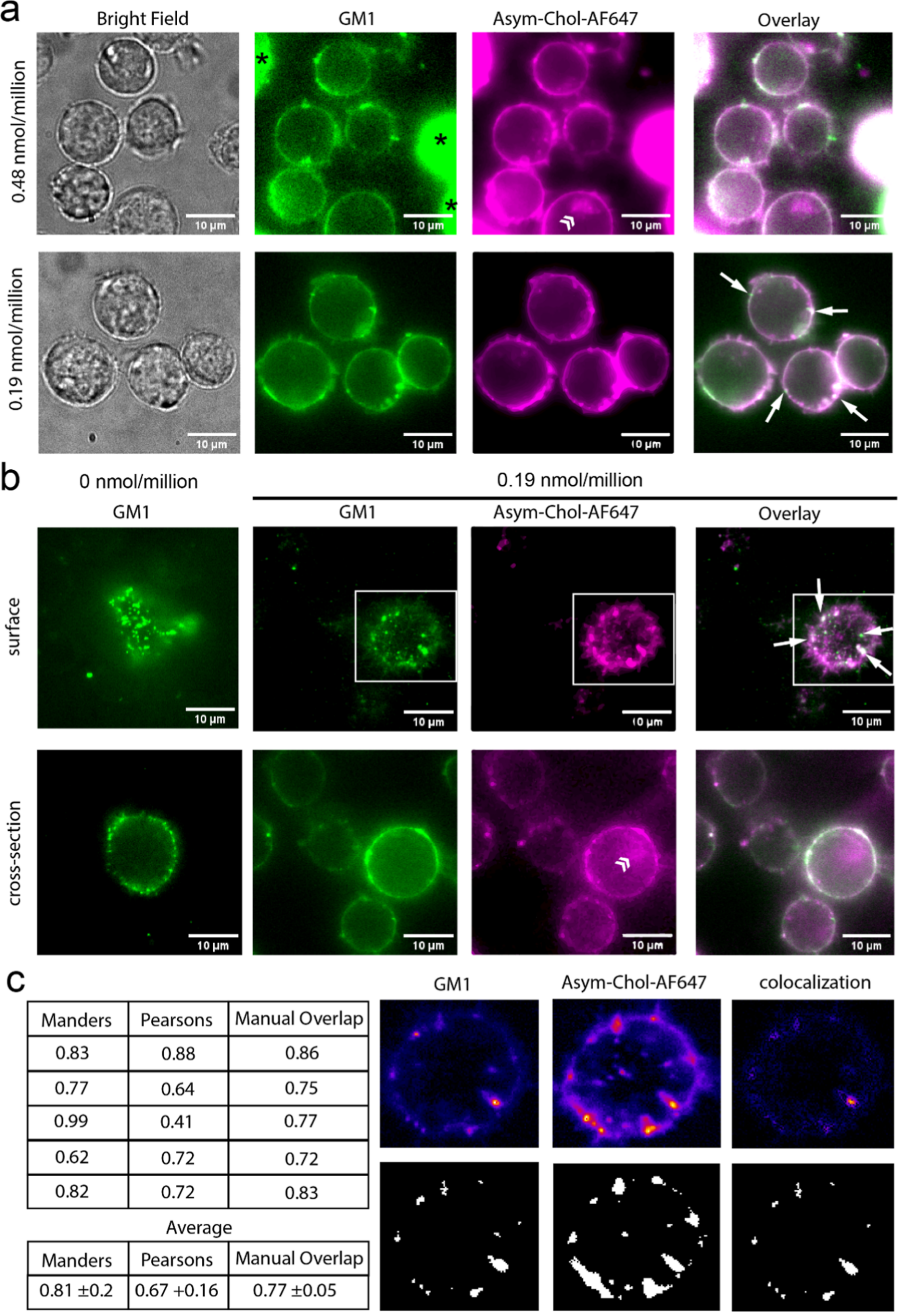
Live cell confocal microscopy of AsymPol-Chol-AF647-labeled JLat 9.2 T cells. (a) Cross sections of JLat 9.2 T cells loaded with 0.48 nmol/million (top panels) and 0.19 nmol/million cells of AsymPol-Chol-AF647 (bottom panels) where AsymPol-Chol-AF647 is shown in magenta (magenta) and the lipid-raft marker GM1 in green. * indicates dead cells, ≫ indicates internalized AsymPol-Chol-AF6 to Golgi-like structures, and white arrows indicate lipid raft structures. (b) Surface (top panels) and cross sections (bottom panels) of JLat 9.2 T cells loaded with (right side images) and without (far left) AsymPol-Chol-AF647. Images were acquired with a Nikon Ti2 microscope equipped with a confocal rescan module. All cells were imaged within 30 min of preparation for DNP. (c) Colocalization analysis of 0.19 nmol/million PA-loaded JLat 9.2 T cells. Colocalization was quantified using Coloc 2 plugin of ImageJ on background corrected images with Costes automatic thresholding. The Manders coefficient is given as a measure of GM1-positive pixels that colocalize with PA + pixels. Pearson’s analysis indicates the intensity correlation between GM1 and PA staining. Colocalization was also determined using the binary overlap method on background subtracted images manually thresholded. Example images used in manual colocalization are shown for the region of interest indicated by the box in (b). Analysis of additional images used for quantification are given in Supporting Information-5.

Further dilution of the PA to 190 μM (0.19 nmol/million cells) improved cell survival to 95.8% live cells, which is comparable to that of untreated cells (Supporting Information-3). Plasma membrane staining with GM1 increased in intensity with improved discretization of GM1-positive clusters, suggesting improved lipid raft integrity. Asym-Chol-AF647 may be expected to show perturbed phase partitioning behavior compared to native cholesterol as seen for many fluorescent cholesterol derivatives in giant unilamellar vesicles (GUVs),^37,38^ but it could be readily established that this PA does indeed partition into lipid rafts with 77% of GM1-positive structures also staining for the PA. Whether the localization of the PA into GM1-positive structures is thermodynamically driven by phase order preferences is unclear. Interestingly, the PA was mostly associated with larger membrane microdomains of >200 nm, while smaller domains remained PA free. Significant staining outside these domains was also observed but largely restricted to the plasma membrane, with some cells displaying Golgi-like staining at longer time periods, as observed at 0.48 nmol/million (Supporting Information-4). To conclude, the PA, AsymPol-Chol-AF647, displays excellent localization to the plasma membrane and has distinct concentration-dependent effects on the integrity of microdomain structures within the plasma membrane.

### In-Cell DNP-NMR with AsymPol-Chol-AF647

We next assessed the DNP enhancements of JLat 9.2 T cells loaded with AsymPol-Chol-AF647, using the same cells from which live-cell confocal imaging was performed but flash-frozen within 5 min of addition of the PA (prior to microscopy analysis). We observed low enhancements of natural abundance ^13^C: ε < 4 at 9.67 nmol/million and ε < 5 at 0.48 nmol/million (Figure 3a,b). Distinct spectral differences were observed between the sample that lysed (9.67 nmol/million loaded) and the intact sample (0.48 nmol/million loaded) (Figure 3d), particularly narrowing of the CH resonances for the former. Those signals are predominantly associated with lipids and, to a lesser extent, carbohydrates and protein Cα atoms. Loading cells with 0.19 nmol/million PA gave greatly improved natural abundance ^13^C enhancements of ε = 10–14 (Figure 3c).

**Figure 3:**
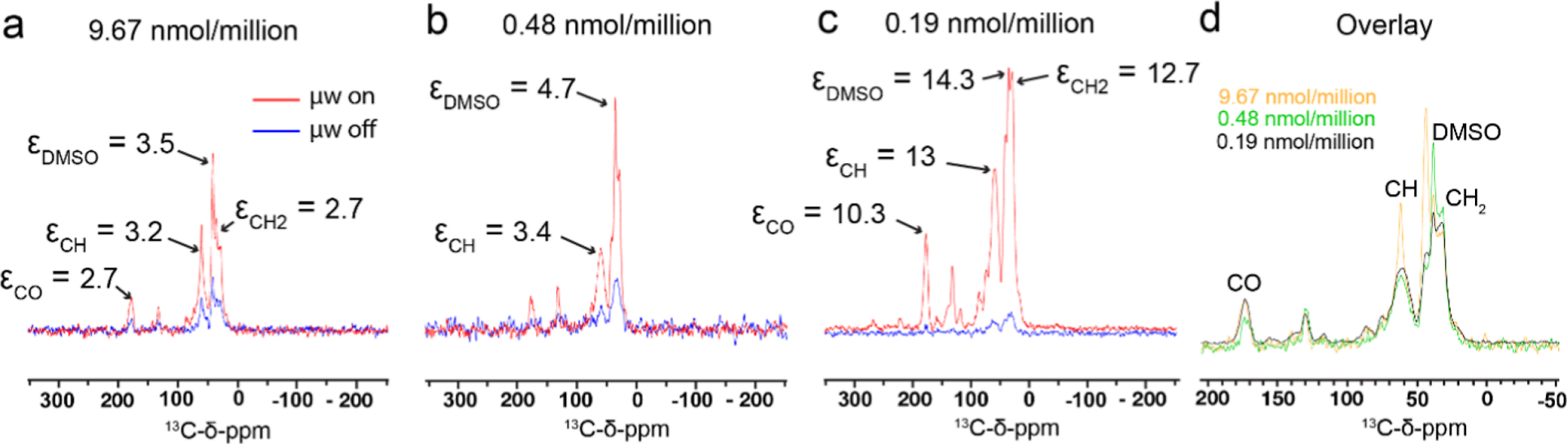
DNP enhancements of ^13^C cross-polarization-detected AsymPol-Chol-AF647-labeled JLat 9.2 T cells. JLat 9.2 T cells loaded with (a) 9.48 nmol/million AsymPol-Chol-AF647, (b) 0.48 nmol/million AsymPol-Chol-AF647, (c) 0.19 nmol/million AsymPol-Chol-AF647. (d) Overlay of the microwave-on data shown in (a–c). The data was acquired at 9.4 T with 9 kHz MAS at 86 K without microwaves and at 89 K with microwaves; 128 transients were collected. JLat 9.2 T cells were loaded with the indicated amounts of the bimodal cholesterol, cryopreserved with 10% DMSO-*d*_6_ and flash-frozen in liquid nitrogen within 5 min of the addition.

Radical concentrations for DNP NMR of biomolecular samples are usually in the 5–40 mM range.^39–44^ If the radical was evenly distributed throughout the cellular sample, the concentration of the PA would be 9.67 mM, 0.48 mM, and 0.19 mM. However, given that the plasma membrane accounts for 1% of the cell volume and that the localization of the PA is highly restricted to the plasma membrane, at least at 0.48 and 0.19 nmol/million loadings, we can estimate the local concentration of AsymPol-Chol-AF647 in the plasma membrane to be 48 and 19 mM, respectively. The residual AF647 fluorescence in the supernatant was measured to be 100 and 39 μM, respectively, using UV–vis spectroscopy. Correcting for this solvent-dissolved Asym-Chol-AF647, we estimate the membrane concentration of the probe to be 47.9 and 18.96 mM, respectively. The low enhancements at 0.48 nmol/million are probably the result of fast *T*_2,e_, due to intermolecular electron–electron dipolar couplings between biradicals that would be expected at 48 mM local concentration. The equally low enhancements at 9.67 nmol/million suggest that even though the cells are lysed at this concentration, the local concentration is likely much higher than the expected 9.67 mM for an even distribution of radical, suggesting an inhomogeneous distribution even under these conditions. The estimated PA concentration at the plasma membrane is high for typical water-soluble PAs such as AsymPol-POK^32^ and AMUPol.^45^ The enhancements at 0.19 nmol/million in cells (19 mM at the plasma membrane) are half that reported for 10 mM AsymPol (under ideal conditions in DNP juice) at the same magnetic field (9.4 T). However, given the nonuniformity of the distribution of AsymPol-Chol-AF647 in cells, the local enhancement at the plasma membrane is unclear. Since the region that the radical directly hyperpolarizes represents only around 1% of the total cellular volume, much of the sample is likely not contributing to the microwave on signal; thus, the local enhancement in the vicinity of the radical likely exceeds ε = 14.

In general, DNP enhancements are observed when spin hyperpolarization, from the free electrons of the radical, is transferred to nearby nuclei that are hyperfine coupled to the radical. These hyperpolarized nuclei can then transfer their polarization to nearby nuclei through spin diffusion, which strongly affects the enhancement value ε. Spin diffusion is a homonuclear process (under most conditions), and its dynamics are determined by the dipolar coupling between homonuclear spins. Thus, the closer the nuclei are to each other, the faster the transfer of polarization and a concomitant faster buildup of the NMR signal to a maximum. However, the presence of the radical also impacts the rate in which hyperpolarization builds up on nearby nuclear spins and thereby the maximum achievable enhancement (ε), by affecting the rates of nuclear relaxation. The result is that the buildup of hyperpolarization on nuclei near the radical will be different from that of nuclei that are far from the radical. For nuclei that are close enough to the radical to be hyperfine-coupled (typically 20–30 Å radius), they can receive hyperpolarization either directly from the electron or through spin diffusion from nearby hyperpolarized nuclei. For nuclei that are further away from the radical and have no hyperfine interaction with the electrons, they can receive hyperpolarization only through spin diffusion. We will refer to nuclear spins in these two regions as hyperfine-coupled nuclei and uncoupled nuclei, respectively.

It is possible to estimate how far the generated hyperpolarization will travel from the radical before reaching 36% of its maximal value by calculating the diffusion length using eq 1:

1 $$L=\sqrt{DT_{1,uncoupled}}$$

Where the spin diffusion coefficient (*D*) is the rate at which polarization is transferred between nuclei and *T*_1,uncoupled_ is the intrinsic spin–lattice relaxation rate of the receiving nuclei in the absence of radicals (undoped). *T*_1,uncoupled_ was directly measured on a sample of JLat 9.2 T cells without a radical, giving an average undoped *T*_1,uncoupled_ of 25 s (Supporting Information-6) and a diffusion length (*L*) of 160 nm. This assumes a spin diffusion coefficient of around 1 × 10^–3^ μm^2^ s^–1^, a typical value for samples containing a high concentration of protons.^46^ The enhancement of hyperfine-coupled nuclei (ε_local_) in 0.19 nmol/million loaded cells can now be estimated to be 136, using the measured global enhancement (all spins) of 14, according to eq 2:

2 $$\varepsilon_{global}=1+\left(\varepsilon_{local}-1\right)3\frac{L}{r}$$

assuming an average cell radius (*r*) of 5 μm (determined from the bright-field imaging in Figure 2) and the calculated diffusion length (*L*) of 160 nm. This value is comparable to the range reported for AsymPol derivatives AsymPol-POK^32^ and AsymPol-TEK^47^ in a homogeneously distributed sample in a glassy matrix. Without a spectrally resolved resonance known to be hyperfine-coupled to the radical, the actual enhancement is unclear for hyperfine-coupled nuclei. However, we know the dimensions of the region that contains the hyperfine-coupled spins (plasma membrane) are much smaller than the region that contains the uncoupled spins (the rest of the cell), and thus, the expectation is that the global enhancements should be small. In fact, this is the case for 0.48 nmol/million loaded cells. Using the measured global enhancement of 4 and the same diffusion length of 160 nm, eq 2 gives ε_local_ of 30 at 0.48 nmol/million PA. Thus, the concentration of 0.19 nmol/million PA loaded cells seems ideal for maximizing polarization transfer efficiency while minimizing competing relaxation effects.

The plasma membrane localization of the PA together with the significant enhancement opens up the possibility that hyperpolarization dynamics can be measured and used to confirm the microscopy data, namely, that the cells are 5 μm spheres with a small layer of radical around the surface (core–shell model). We therefore measured the signal buildup as a function of polarization time with and without microwave irradiation. At 0.48 nmol/million, the signal buildup tends toward faster rates with microwave irradiation (Figure 4a), averaging a *T*_B,ON_ of 7.2 s and *T*_B,OFF_ of 8.55 s. This is expected when the PA is inhomogeneously distributed in the sample,^29^ as observed with confocal imaging (Figure 2).

**Figure 4:**
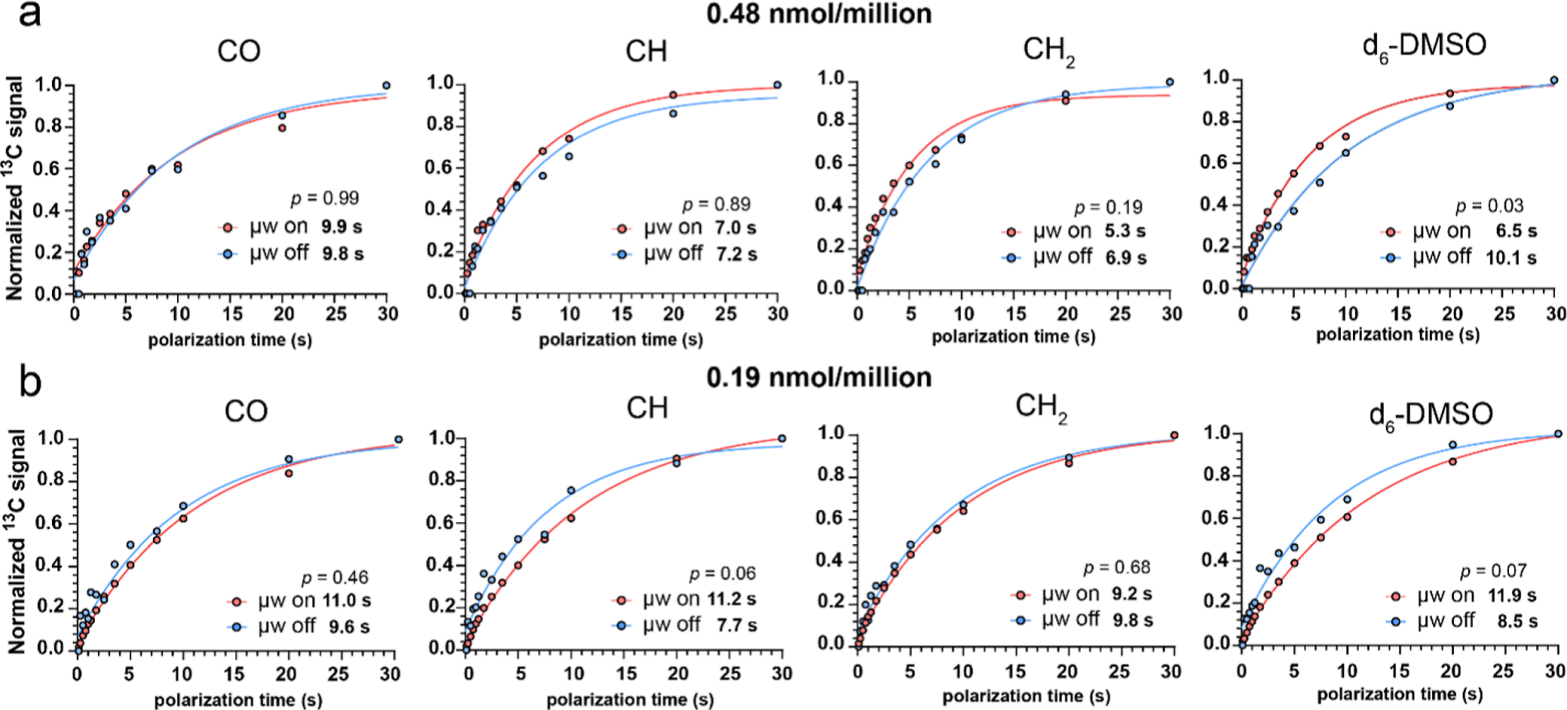
T_B_ profiles of AsymPol-Chol-AF647 loaded T cells. (a) 0.48 nmol/million AsymPol-Chol-AF647 and (b) 0.19 nmol/million AsymPol-Chol-AF647. Data was acquired using a saturation recovery experiment with cross-polarization enhanced detection of ^13^C nuclei with 9 kHz MAS at 9.4 T external magnetic field at 86 K without microwaves and 89 K with microwave irradiation. The data were fit to a single exponential buildup function as described in the methods and the buildup rate constant (1/*T*_B_) determined. The significance of the difference between microwave on and off buildup rates was determined using an F-test of the difference in 95% confidence intervals of the fitted rate constant 1/*T*_B_. Differences were considered significant when the probability (*p*) is ≤0.05. Signal buildup data for 9.67 nmol/million PA loading is shown in Supporting Information-7.

When cells are loaded with 0.19 nmol/million PA, the signal builds up slower with microwaves than without (Figure 4b), averaging a *T*_B,ON_ of 10.8 s vs *T*_B,OFF_ of 8.9 s. The fact that *T*_B,ON_ is now slower than *T*_B,OFF_ indicates that the polarization transfer dynamics (either from the electron to hyperfine-coupled spins or between nuclei) has changed compared to the faster *T*_B,ON_ value measured at 0.48 nmol/million PA. *T*_B_ values without microwaves were comparable to those measured at 0.48 nmol/million PA.

In general, the expectation is that the signal buildup is faster in the presence of microwave irradiation due to the fast transfer of spin-polarization from the microwave-polarized electrons to hyperfine-coupled nuclei.^29^ Polarization then spreads to the rest of the hyperfine-coupled nuclei and into the uncoupled nuclei through spin diffusion. For the signal buildup to slow with microwave irradiation, the rate of transfer of polarization between hyperfine-coupled nuclei or between the hyperfine-coupled nuclei and uncoupled nuclei must be slower than that between the uncoupled nuclei themselves. This could be due to a lower diffusion coefficient within the region of hyperfine-coupled nuclei compared to the uncoupled nuclei due to the accumulation of deuterated solvent in the vicinity of the radical. Alternatively, the radical concentration could be very low, relative to the local ^1^H concentration, resulting in a small number of polarization transfer events per unit time. Both scenarios yield the same result, namely, that the signal buildup is limited by spin diffusion in the vicinity of the radical.

To better understand the potential sources of slower hyperpolarization buildup with microwave irradiation, we simulated hyperpolarization dynamics utilizing the theoretical framework established by Emsley and colleagues, based on well-described diffusion phenomena, for the description of hyperpolarization transfer over time.^25,29,30^ The model shown in Figure 5a was used to simulate the distribution of hyperpolarization. We model a sphere in which the radical coats the surface based on the microscopy data. The length of the uncoupled bulk is set to the measured cell radius of 5 μm. The intrinsic *T*_1_ of these uncoupled spins was also set to the measured value of undoped cells of 25 s. We then assume the spin diffusion coefficient is similar to that measured for other proton-rich biological samples^46^ of 1 × 10^–3^ μm^2^ s^–1^. We then use this model to fit the experimental data to estimate the local enhancement (ε_local_) and local nuclear relaxation rate (*T*_1,coupled_).

**Figure 5:**
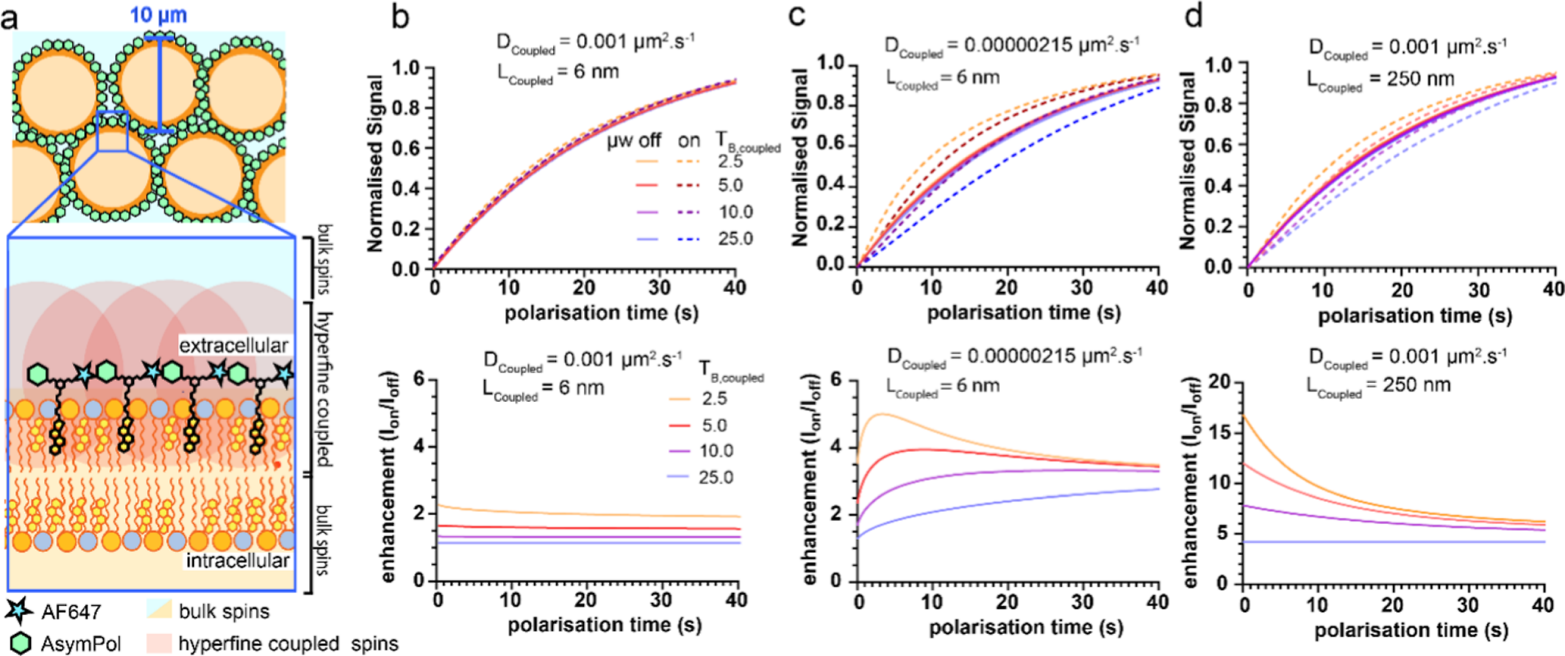
A model system for analyzing spin diffusion and simulated hyperpolarization behavior over time. (a) Schematic of the model system used in the simulations. The volume that contains nuclei that are hyperfine-coupled to the AsymPol radical is shaded in red. Uncoupled nuclear spins (blue and orange shade) are nuclei that are not hyperfine-coupled to the radical. The PA was also restricted to the plasma membrane, as indicated by the microscopy. We additionally presume that the PA is largely restricted to the outer leaflet of the membrane due to the 4 negatively charged sulfonate groups of the AF647 as seen for similarly sulfonated diacylglycerol derivatives.^48^ (b) The effect of varying *D*_coupled_ on the signal buildup behavior. (c) The effect of varying *D*_coupled_ on the enhancement behavior. (d) The effect of increasing the length of the coupled region to 250 nm (*L*_coupled_). Simulated signal buildup curves with microwaves on (dashed lines) and microwaves off (solid lines). Simulations were done using a MATLAB code published by Pinon A et al.^29^ with the following parameters: *T*_1,uncoupled_ = 25.0 s, spin diffusion coefficient of the uncoupled nuclei (*D*_uncoupled_) = 0.001 μm^2^ s^–1^, *L*_uncoupled_ = 5.0 μm, *L*_coupled_ = 6 nm (except in d) where it is 250 nm), and *L*_border_ between hyperfine-coupled nuclei and uncoupled nuclei spins = 2 nm and ε_local_ = 30. Parameters that were varied are given in the figure.

Within the bounds of our 5 μm core–shell spherical model, the signal buildup becomes slower with microwave irradiation when *T*_1,coupled_ ≈ *T*_1,uncoupled_. Under these conditions, the enhancement becomes negligible, even if the local enhancement is set to a value > 400, inconsistent with the ε_global_ = 14 value experimentally measured (Figure 5b). If we introduce a difference in spin diffusion coefficients between the coupled and uncoupled regions, we can better reproduce the observed phenomena where the microwave on signal buildup becomes significantly slower, particularly when *T*_1,coupled_ ≈ *T*_1,uncoupled_. Furthermore, the enhancement is larger, increasing at early time points, and is scalable with a larger local enhancement (Figure 5c and Supporting Information-8). On the other hand, if instead the length of the coupled region (*L*_coupled_) is increased to 250 nm (up from 6 nm), the signal buildup also becomes slower with microwave irradiation. The enhancements are significantly higher again but decrease at early time points (Figure 5d). Therefore, the enhancement behavior at early time points is informative of the relative magnitude of *T*_1,coupled_, and relatedly, whether spin diffusion may be limited within the vicinity of the radical or if the size of the coupled region might be larger than assumed.

Plotting the enhancement buildup, (the ratio of ^13^C signal $\frac{\mu won}{\mu woff}$ ) as a function of time, we observed an increasing enhancement buildup at 0.19 nmol/million PA (Figure 6b), indicating that the polarization of hyperfine-coupled nuclei might be either spin-diffusion limited or there is a layer in between the hyperfine-coupled spins and the uncoupled spins.^49^ In contrast, at 0.48 nmol/million, the enhancement decreases at early time points or is relatively flat, indicative that the maximum enhancement is likely to be independent of spin diffusion processes and that *T*_1,coupled_ < *T*_1,uncoupled_ (Figure 6a). The size of the coupled region may also be larger than the assumed value of 6 nm in this case.

**Figure 6:**
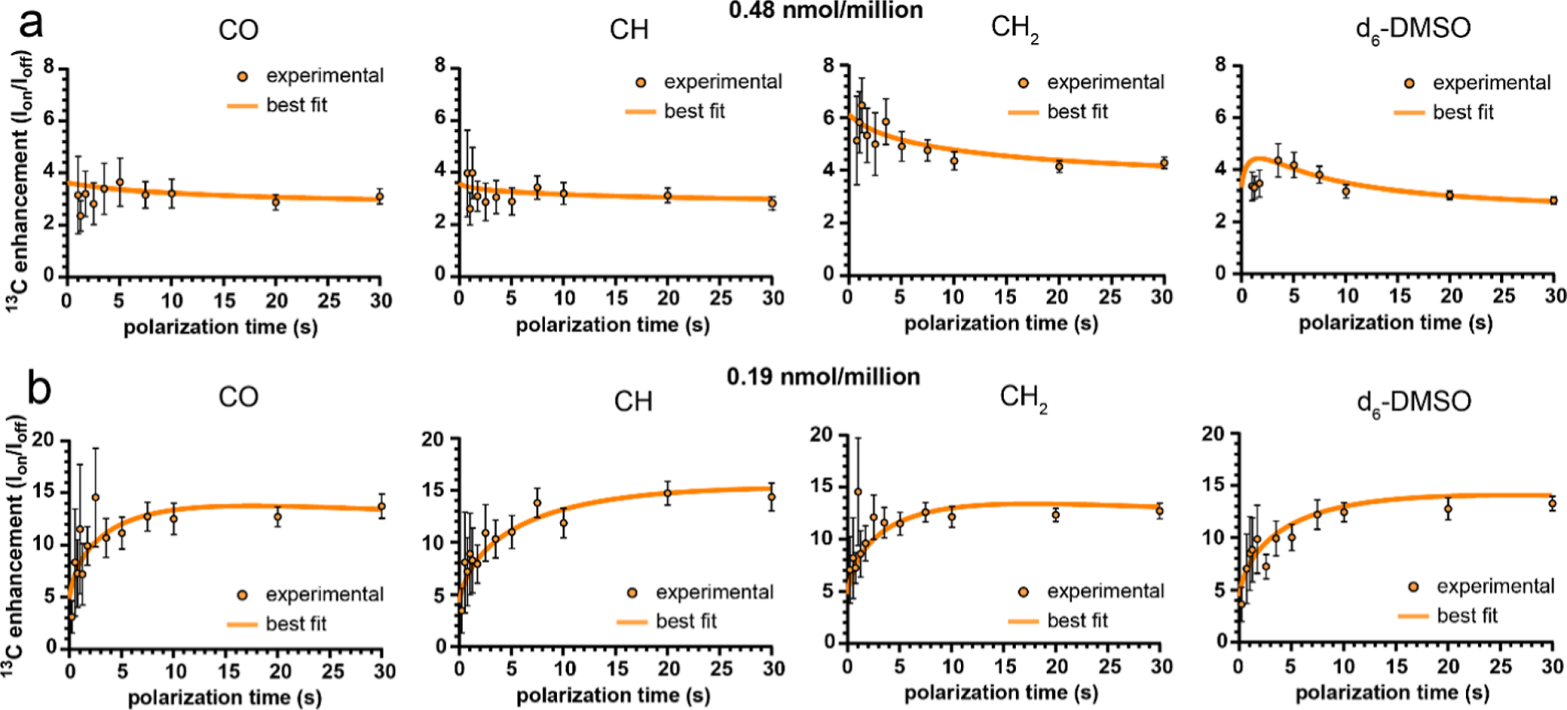
Enhancement buildup curves of JLat 9.2 T cells loaded with AsymPol-Chol-AF647. ^13^C enhancements are calculated as the ratio of the intensity of the indicated ^13^C signals with (*I*_ON_) and without (*I*_OFF_) microwave irradiation as a function of time. (a) Cells loaded with 0.48 nmol/million AsymPol-Chol-AF647. (b) Cells loaded with 0.19 nmol/million AsymPol-Chol-AF647. Error bars indicate the propagated error of the peak intensity. Experimental data was fit (orange line) using the MATLAB code published in Pinon. A. et al.^50^ using the following model parameters: *T*_1,uncoupled_ = 25.0 s, spin diffusion coefficient of the uncoupled nuclei (*D*_uncoupled_) = 0.001 μm^2^ s^–1^, *L*_uncoupled_ = 5.0 μm, *L*_coupled_ = 6 nm, and *L*_border_ between hyperfine-coupled nuclei and uncoupled nuclei spins = 2 nm. Additional fitted parameters are given in Table S1. Data for 9.67 nmol/million loaded cells is given in Supporting Information-9.

The experimental enhancements for both 0.19 and 0.48 nmol/million PA loaded cells could be fit to the 5 μm sphere model shown in Figure 5a using the model parameters discussed and fitting for the local enhancement and *T*_1,coupled_ (Table S1). However, neither could be fit simultaneously with the signal buildup data shown in Figures 4 (Supporting Information-10), although the general trends were reproduced. Enhancement data at 0.19 nmol/million was best fit with values for *T*_1,coupled_ within the range of the experimentally measured *T*_B,ON_ values (Table S1) and the large estimates for ε_local_. These values reproduced the slower signal buildup with microwave irradiation but only when a large difference in diffusion coefficients between the coupled and uncoupled nuclei was introduced into the model. In contrast, experimental enhancement buildup data at 0.48 nmol/million was best fit to *T*_1,coupled_ values that were faster than the experimentally measured *T*_B,ON_ values and to ε_local_ values that were larger than those estimated from the global enhancements. At 0.48 nmol/million PA, a homogeneous diffusion coefficient for CO and CH_2_ resonances was sufficient to fit the data, with a smaller local spin diffusion coefficient required for DMSO-*d*_6_ and CH resonance enhancements (Table S1). We note that the DMSO-*d*_6_ and CH resonances both displayed slower microwave on signal buildup kinetics while CO and CH_2_ resonances showed either no difference or a faster buildup with microwave irradiation, consistent with the fitted parameters for 0.19 nmol/million loading. Furthermore, fitting with a larger uncoupled region (>6 nm) did not improve the fits.

Fitting the characteristic increasing enhancement at early time points in 0.19 nmol/million PA loading was dependent on assuming a spin diffusion gradient between the coupled and uncoupled spins, as this behavior could not be reproduced when a homogeneous value between the coupled and uncoupled spins was considered (Figure 5c and Supporting Information-11). If the length or size of the coupled region is increased, equating to a scenario in which radical is present in the extracellular space or has flipped to the inner leaflet of the membrane, a decreasing enhancement at early time points is observed. The early behavior is sensitive to the length of the coupled region, with an increase to 50 nm from 6 nm sufficient to generate a curve that is inconsistent with the experimental enhancement (Supporting Information-11). However, if *T*_1,uncoupled_ is set to be equivalent to *T*_B,OFF_, the curves become less sensitive to the length of the coupled region (Supporting Information-11b), generating curves that increase at early time points. As the length of the coupled region increased from 6 to 500 nm, the fitted local enhancement decreased from ε = 161 (when *T*_1,uncoupled_ was equal to the measured value of 25.0 s) to ε = 55 when *T*_1,uncoupled_ was equal to *T*_B,OFF_ (8.3 s) (Supporting Information-11b). However, once again, the early behavior of the enhancement buildup curves could only be reproduced when a difference in spin diffusion coefficients between the coupled and uncoupled regions is introduced into the model.

## Discussion

In this study, we have demonstrated the synthesis of a new multimodal cholesterol molecule, dubbed AsymPol-Chol-AF647, that can be used for plasma membrane targeted in-cell DNP-NMR studies. This PA enabled the determination of its localization within cells by fluorescence microscopy, as well as providing significant DNP enhancements of NMR signals originating from the cell surface. Analysis of the hyperpolarization dynamics revealed a more complex buildup behavior than would be expected from the simple 5 μm spherical model implied by the microscopy data. This result suggests that the hyperpolarization buildup dynamics are sensitive to the local cellular environment and could potentially report on more complex cellular architectures such as lipid rafts. We take the first step toward utilizing the kinetics of hyperpolarization distribution to investigate cellular architecture.

The hydrophobic cholesterol moiety in AsymPol-Chol-AF647 drives its incorporation into the cell membrane to the extent that the cells lyse at high concentrations. This affinity for the membrane also facilitates efficient cellular loading with AsymPol-Chol-AF647 at lower radical concentrations. The addition of the fluorophore to cholesterol also allows us to be confident that the AsymPol-Chol-AF647 is restricted to the plasma membrane and is unlikely to significantly flip to the inner leaflet, similarly to other fluorophore-labeled lipids that show restricted localization to the outer leaflet.^48^ However, the PA seems to perturb the microdomain structure of the membrane, at the concentrations tested, although the perturbations were greatly reduced at 0.19 nmol/million PA, with distinct GM1-positive microdomains visible (identified as lipid rafts^51^) compared to 0.48 nmol/million PA. GM1 staining was weak and less discrete indicating a disruption to lipid raft integrity at 0.48 nmol/million.^38^ The contribution of ordered-phase preference of the PA to raft association in cells is of interest, as it may explain the perturbing nature of high concentrations of the PA on the morphology of GM1-positive domains. This is of particular interest, given the scale disparity between the radical concentration needed for efficient DNP and the fluorophore concentration needed for microscopy studies. However, we demonstrate the compatibility of these visualization modes.

Based on the fluorescence images, the dimensions for the region of radical coupled nuclei and that of uncoupled nuclei could be established, enabling us to generate a simplified model for simulating the dynamics of hyperpolarization buildup within the sample. We could model the hyperpolarization buildup behavior at 0.48 nmol/million using the established model, in which the radical is localized to a very small region at the periphery of a relatively large sphere. We did not account for the 15% dead cells observed at 0.48 nmol/million PA, which would be expected to exhibit homogeneous buildup dynamics independent of microwave irradiation (similar to that observed at 9.67 nmol/million). Thus, the presence of these cells may dampen the difference in polarization buildup rates between those with and without microwaves. Furthermore, this model and the calculated diffusion lengths fit a local enhancement estimation of ε = 30.

However, at 0.19 nmol/million, this same model estimates a local enhancement of 136 for the global measured enhancement of 14. Such a value would be in the range of the largest values reported for any AsymPol derivative at 9.4 T,^32,47^ indicative of close to ideal DNP conditions such as the radical and ^1^H concentration. The enhancement behavior could be fit to our simple core–shell spherical model, using the experimental parameters and the predicted local enhancements at both 0.48 nmol/million and 0.19 nmol/million but only if a difference in spin diffusion coefficients between the coupled and uncoupled regions is introduced into the model. These parameters were also required to reproduce the slower signal buildup when microwaves are on compared to that when microwaves are off, observed at 0.19 nmol/million PA. However, the enhancement buildup data could not be simultaneously fit with the signal buildups (*T*_B_). These parameters simulate much larger *T*_B_ values with and without microwaves than those experimentally measured.

The early behavior of the system appears sensitive to the length or size of the coupled region but only if *T*_1,uncoupled_ is long (>20 s). When *T*_1,uncoupled_ is shorter, the enhancement buildup becomes less sensitive to the length of the coupled region. However, even when significant allowances are made for variations in the length of the radical coupled region or the value of *T*_1,uncoupled_, the enhancement and signal buildup data could not be simultaneously fit. Together the data indicate that the hyperpolarization dynamics are sensitive to local cellular complexity that the simple model suggested by the microscopy does not capture. The best fit data suggest that this in part might be accounted for by differences in the spin diffusion coefficient between coupled and uncoupled spins. The data therefore suggests that cellular sources of perturbations to the hyperpolarization dynamics need to be considered, such as the role of deuterated solvents and their inhomogeneous cellular distributions^52–54^ in determining local spin-diffusion coefficients. The effects of highly methylated cellular components^55–58^ such as cell membranes which comprise 40–60% phosphatidylcholine and sphingomyelin, whose headgroups each contain three methyl groups also need to be considered.^59–62^ In this context, the contribution of ^1^H atoms from the polarizing agent itself to local spin diffusion coefficients will also be a contributing factor in determining the hyperpolarization dynamics, or the ratio of radical-to-^1^H atoms being below a threshold, such that few ^1^H atoms are directly hyperpolarized per unit time. The latter scenario limits the buildup of hyperpolarization to nuclear spin diffusion rates and not electron to nuclear transfer rates. The difference between these scenarios cannot be captured by the current theoretical model for hyperpolarization distributions. These possibilities highlight the exciting potential of these probes for future studies of cells at both the atomic scale and nanoscale with nanoscale analysis afforded by correlation of DNP-NMR and microscopy.

These findings likely reflect the uncertainty of the *T*_1_ values within the coupled and uncoupled regions of the sample as well as our assumptions made for the spin diffusion coefficient, which we have no a priori knowledge of. However, the spin diffusion coefficient could be determined if the *T*_1_ values and local enhancements could be directly measured. This requires the spectroscopic resolution of hyperfine-coupled nuclei from the uncoupled nuclei. This could be achieved with an AsymPol-Chol-AF647 molecule that contains a distinct ^13^C or ^19^F resonance in addition to the labeling of uncoupled nuclei or a portion of uncoupled nuclei with an equally distinct resonance. Such a labeling scheme would then allow accurate determination of the local enhancements and a greater understanding of the distribution of hyperpolarization within cells using localized probes. This kind of information is critical for establishing the use of these PAs, not just in local enhancements for regions of interest, but additionally in providing a means for understanding the local geometry and relative spatial organization of labeled cellular components and complexes in cellular populations. This has significant implications for enabling meaningful fitting of the hyperpolarization buildup data to discrete raft domain sizes together with cholesterol derivatives with improved raft localization behavior. Such information could be used to characterize the change in raft size and organization over a variety of cellular conditions in a whole population of cells, providing a complementary analysis to that of individual cells, which is largely the methodology employed by fluorescence microscopy studies. This is a particularly compelling possibility for the study of immune cells whose physiological outcomes are the result of distinct nanoscale changes in lipid raft organization^63,64^ and thus the potential of these bimodal probes is clear.

## Conclusion

We have developed a cholesterol-based PA that integrates fluorescence imaging with in-cell DNP-NMR, allowing direct visualization of radical localization while simultaneously providing significant enhancement of the membrane DNP signals. This approach has the potential to bridge micrometer-scale subcellular localization with nanometer-scale hyperpolarization behavior. Ultimately, such probes, combined with the measurements of hyperpolarization buildup dynamics, together with improved lipid-raft targeting, could open a path toward characterizing lipid-raft size and organization under different cellular conditions across whole cell populations, complementing fluorescence-based methodologies.

## Supplementary Material



## Notes

Experimental materials and methods including: synthetic methods, including ^1^H and ^13^C NMR data and AT-IR characterization of all compounds synthesized; cell survival analysis; *T*_1_ buildup analysis for undoped cell samples; additional confocal images of Asym-Chol-AF647 localization and colocalization analysis; simulated *T*_B_ fits to accompany the enhancement fits; *T*_B_ data and enhancement data for cells loaded with 9.67 nmol/million PA; and additional simulations of enhancement and signal buildups (PDF)

## Abbreviations

DNP: dynamic nuclear polarization
NMR: nuclear magnetic resonance
GM1: monosialotetrahexosylganglioside
AF647: AlexaFluor-647
DMSO: dimethylsulfoxide
