# Supplementary material for: Bimodal Cholesterol for Correlative In-Cell DNP Solid-State NMR and Confocal Microscopy of the Plasma Membrane
Source: J Am Chem Soc. 2026 Apr 9;148(15):15618–29. doi: 10.1021/jacs.5c20070 (PMC13107443; doi:10.1021/jacs.5c20070)
Supplement: Supplementary file 1 [file ja5c20070_si_001.pdf]

Supporting Information

Bimodal cholesterol for correlative in-cell DNP solid-state NMR and confocal microscopy of the plasma membrane

Sarah A. Overall*[1], Ancy Wilson[2], Dorothea Pinotsi[3], César Själsjö[4], Snorri Th. Sigurdsson[2] and Alexander B. Barnes*[1]

[1]Institute of Molecular Physical Science, ETH Zurich, 8093 Zurich, Switzerland

[2]Science Institute, University of Iceland, Dunhagi 3, 107 Reykjavik, Iceland

[3]Scientific Center for Optical and Electron Microscopy, ETH Zurich, 8093 Zurich, Switzerland

[4]Swedish NMR center, University of Gothenburg, 40530 Gothenburg, Sweden

*Correspondence: soverall125@gmail.com, snorrisi@hi.is and abarnes@ethz.ch

Table of Contents

SI-1: Synthetic Methods…………………………………………………………………………..2

SI-2: Materials and Methods…………………………………………………………………………21

SI-3: Cell survival after loading with tripod-cholesterol ……………………………………………24

SI-4: Time course of Asym-Chol-AF647 internalization ……………………………………………25

SI-5: Colocalization analysis by binary overlap of JLat 9.2 T cells loaded with 0.19 nmol/million Asym-Chol-AF647 (PA). ……………………………………………………………………………26

SI-6: T_1 buildup for JLat 9.2 T cells in the absence of radical. ……………………………………27

SI-7: Simulated T_B fit to experimental polarization buildup data in 9.67 nmol/million PA loaded cells ……………………………………………………………………………………………28

SI-8. Increasing the local enhancement scales the global enhancements when the local diffusion coefficient is smaller than the bulk diffusion coefficient………………………………………………*29*

SI-9: Enhancement buildup curves of JLat 9.2 T cells loaded with 9.67 nmol/million AsymPol-Chol-AF647…………………………………………………………………………………………30

Table S1: Best Fit parameters for ^{13}C enhancement buildups……………………………………31

SI-10: Simulated signal buildup curves using the best fit parameters for the enhancement data shown in **Figure 6**……………………………………………………………………………………32

SI-11: Influence of $T_{1,intrinisc}$ and length of the uncoupled region on the enhancement buildup fitting parameters……………………………………………………………………………………… 33

SI-12: Recycle delay optimization……………………………………………………………………34

References……………………………………………………………………………………………35

SI-1: Synthetic Methods

General materials and methods. All commercially available reagents were purchased from Sigma-Aldrich, abcr Chemicals, AA blocks, AMT, TCI, or Jena Bioscience GmbH and used as received. CH_2Cl_2 and CH_3CN were dried over CaH_2 and freshly distilled before use. All moisture- and air-sensitive reactions were carried out in oven-dried glassware under an inert atmosphere of argon. Analytical thin-layer chromatography (TLC) and preparative TLC purifications were carried out using glass plates precoated with silica gel (0.25 mm, F-254, Silicycle) and compounds were visualized under UV light as well as staining with *p*-anisaldehyde. Column chromatography was carried out using 230−400 mesh silica gel (F60, Silicycle). ^1H and ^{13}C NMR spectra were recorded on an UltraShield 400 MHz Bruker NMR spectrometer. Chemical shifts were reported in parts per million (ppm) relative to the deuterated NMR solvent $CDCl_3$ (7.26 ppm for ^1H NMR and 77.07 ppm for ^{13}C). Commercial grade $CDCl_3$ was dried over molecular sieves prior to use. Radicals show broadening and loss of NMR signals due to their paramagnetic nature and, therefore, NMR spectra of radicals are not shown. CW-EPR spectra were recorded on a MiniScope MS200 spectrometer (Magnettech Germany). Mass spectrometric analyses of all organic compounds were carried out on a high-resolution mass spectrometer with electrospray ionization (ESI-HRMS, Bruker, MicrOTOF-Q) in positive or negative ion mode. The fluorescence spectrum of AsymPol-Chol-AF647 was recorded on Fluoromax-4 spectrofluorometer (HORIBA Scientific) and UV-Vis spectrum on Agilent Cary UV-Vis Multicell Peltier spectrophotometer.

The HPLC analyses and purifications were carried out on an Agilent 1200 HPLC system, with a flow rate of 0.75 mL/min, as follows: Gradient A: solvent A, 0.1% TFA in water; solvent B, CH_3CN; isocratic 4% B for 2 min, 13 min linear gradient to 100% B, 2 min isocratic 100% B, 1 min linear gradient to initial conditions, where it was run for an additional 2 min. Gradient B: solvent A, MeOH; solvent B, CH_3CN; isocratic 4% B for 15 min. Gradient C: the same as gradient A except solvent A, 50 mM HFIP in water; solvent B, CH_3CN, using one of the following columns: Macherey-Nagel Nucleodur C18 Pyramid, Pursuit 5 C8 Pyramid, Pursuit 5 C18 Pyramid.

Abbreviations: TFA, trifluoroacetic acid; MeOH, methanol; HFIP, hexafluoroisopropanol; THF, tetrahydrofuran; BOP, benzotriazol-1-yloxytris(dimethylamino)phosphonium hexafluorophosphate; HOBt, hydroxybenzotriazole; DIPEA, N,N-diisopropylethylamine; EtOH, ethanol; aq, aqueous; EtOAc, ethyl acetate; pet. ether, petroleum ether.

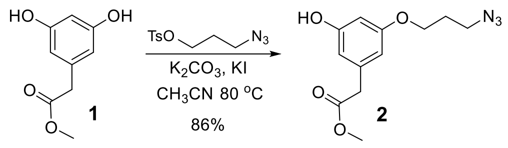

Compound 2. To a solution of compound **1** (200 mg, 1.10 mmol) in CH_3CN (10 mL) were added tosylated azidopropane[1] (84 mg, 0.33 mmol), K_2CO_3 (303 mg, 2.19 mmol), and KI (4 mg, 0.22 mmol) and the solution stirred at 80 °C for 18 h. Satd. aq. $NaHCO_3$ (50 mL) was added, followed by extraction with EtOAC (3 × 30 mL). The combined organic phases were washed with brine (30 mL), dried over Na_2SO_4 and the solvent was removed under reduced pressure. The residue was purified by flash-column chromatography using gradient elution (EtOAC:pet. ether 5:95 to 50:50) to yield compound **2** (75 mg, 0.28 mmol, 86% yield) as a colourless liquid.

<u>TLC</u> (Silica gel, *p*-anisaldehyde, EtOAC:pet. ether 1:1), R_f (**2**) = 0.6

<u>ESI-HRMS (m/z)</u>: calcd. for $C_{12}H_{15}N_3O_4$ $[M+Na]^+$ 288.0955, measured 288.0942

<u>^{1}H NMR</u> (400 MHz, $CDCl_3$) δ 6.37 (dt, *J* = 7.6, 1.8 Hz, 2H), 6.30 (t, *J* = 2.2 Hz, 1H), 6.10 (s, 1H), 3.97 (t, *J* = 5.9 Hz, 1H), 3.70 (s, 3H),3.52 (s, 2H), 3.48 (t, *J* = 6.7 Hz, 2H), 2.04-1.98 (m, 1H) ppm.

<u>^{13}C NMR</u> (101 MHz, $CDCl_3$) δ 172.63, 160.13, 157.24, 136.10, 109.19, 108.07, 101.02, 64.64, 52.44, 48.33, 41.30, 28.79 ppm.

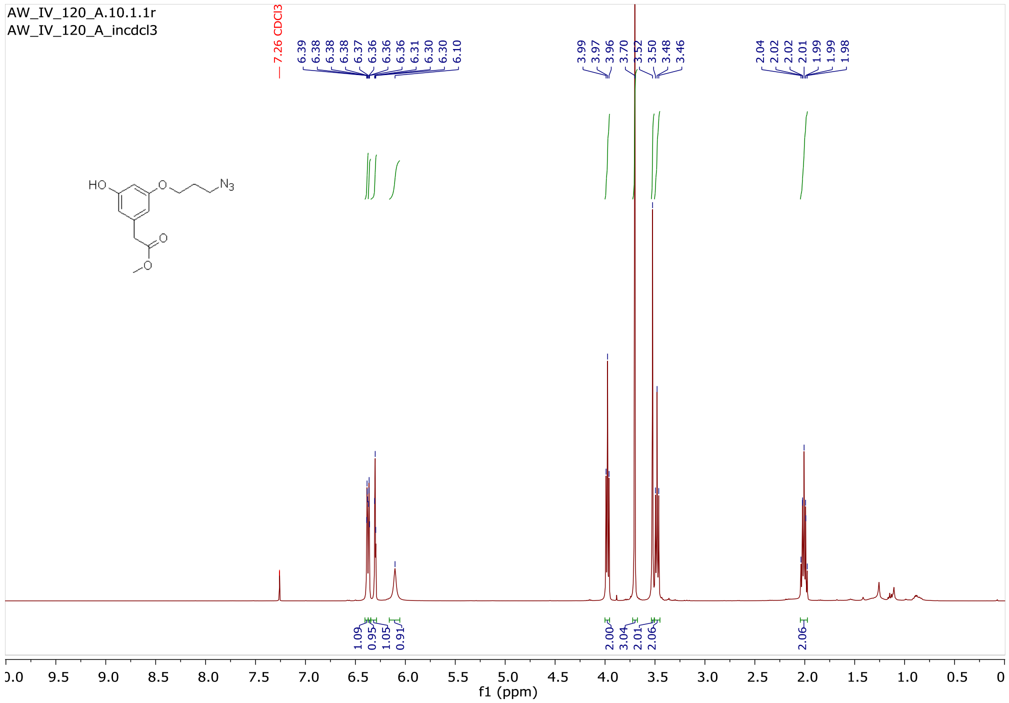

Figure 1. ^{1}H NMR spectrum of **2** in $CDCl_3$.

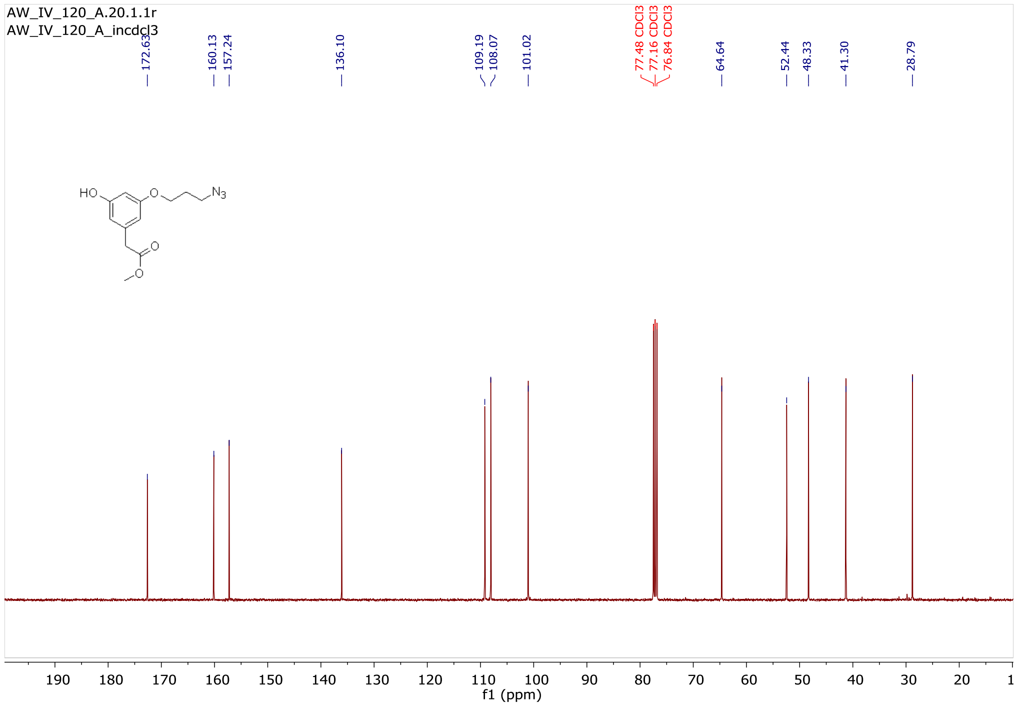

Figure 2. ^{13}C NMR spectrum of **2** in $CDCl_3$.

<u>ATR-IR</u>: 3393, 2094, 1712, 1596 cm^{-1}

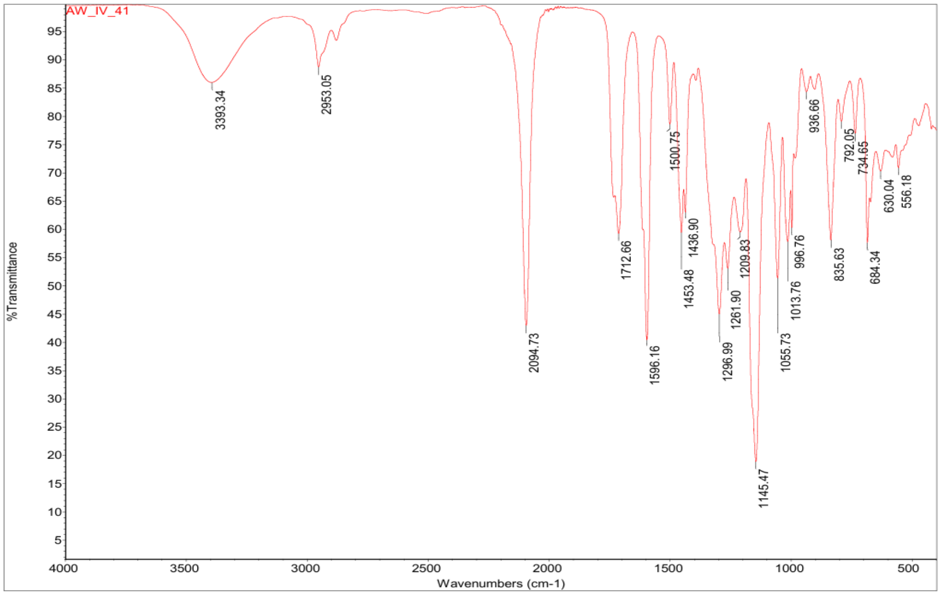

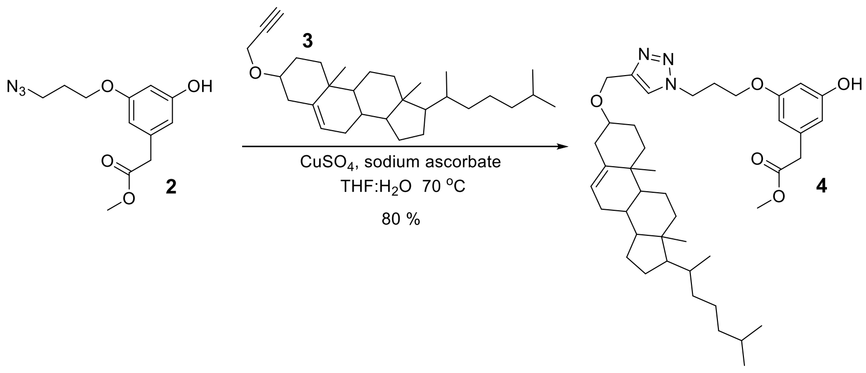

Compound 4. To a solution of compound **2** (113 mg, 0.43 mmol) and (3β)-propargyloxy cholesterol **3**[2] (181 mg, 0.43 mmol) in THF (4 mL) was added a solution of $CuSO_4$ (4 mg, 0.02 mmol) and sodium ascorbate (17 mg, 0.09 mmol) in H_2O (2 mL) and the resulting solution was stirred at 60 °C for 18 h. Satd. aq. $NaHCO_3$ (50 mL) was added, followed by extraction with CH_2Cl_2 (3 × 20 mL). The combined organic phases were washed with brine (20 mL), dried over Na_2SO_4 and the solvent was removed under reduced pressure. The residue was purified by flash-column chromatography using gradient elution (EtOAC:pet. ether 20:80 to 50:50) followed by (MeOH:CH_2Cl_2 5:95 to 10:90) to yield compound **4** (263 mg, 0.38 mmol, 89% yield) as a white solid.

TLC (Silica gel, *p*-anisaldehyde, EtOAC:pet. ether 1:1), R_f (**4**) = 0.2

ESI-HRMS (m/z): calcd. for $C_{42}H_{63}N_3O_5$ $[M+Na]^+$ 712.466, measured 712.4533

^{1}H NMR (400 MHz, $CDCl_3$) δ 7.55 (s, 1H), 6.38 (t, *J* = 1.5 Hz, 2H), 6.27 (t, *J* = 2.2 Hz, 1H), 5.36-5.33 (m, 1H), 4.68 (s, 2H), 4.54 (t, *J* = 6.9 Hz, 2H), 3.93 (t, *J* = 5.8 Hz, 2H), 3.69 (s, 3H), 3.52 (s, 2H), 3.37– 3.29 (m, 1H), 2.43 – 2.33 (m, 3H), 2.28 – 2.21 (m, 1H), 2.04 – 1.92 (m, 3H), 1.88-1.78 (m, 2H), 1.60 – 1.42 (m, 8H), 1.40 – 1.21 (m, 5H), 1.19-1.03 (m, 7H), 0.99 (s, 3H), 0.91 (d, *J* = 6.5 Hz, 4H), 0.86 (dd, *J* = 6.6, 1.8 Hz, 6H), 0.67 (s, 3H) ppm.

^{13}C NMR (101 MHz, $CDCl_3$) δ 172.06, 159.76, 157.59, 145.85, 140.71, 136.39, 123.24, 122.05, 109.63, 107.91, 100.97, 79.33, 64.17, 61.56, 56.90, 56.29, 52.29, 50.28, 47.47, 42.46, 41.31, 39.91, 39.66, 39.10, 37.28, 36.97, 36.33, 35.93, 32.08, 32.02, 29.93, 28.41, 28.37, 28.16, 24.43, 23.97, 22.96, 22.70, 21.21, 19.50, 18.86, 12.00 ppm.

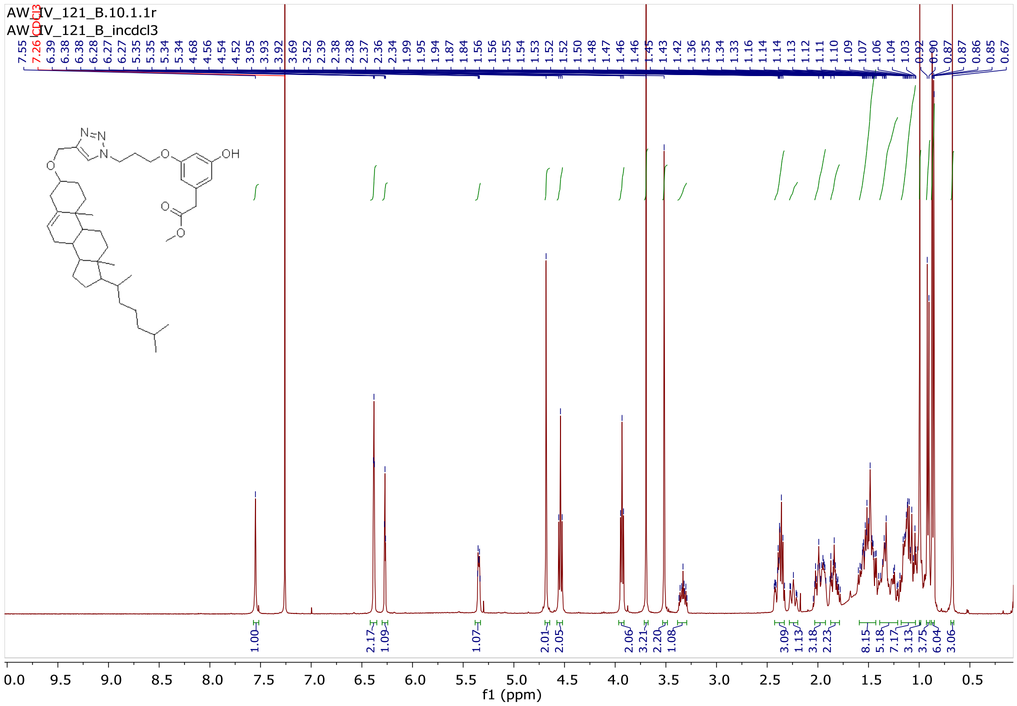

Figure 3. ^{1}H NMR spectrum of **4** in $CDCl_3$.

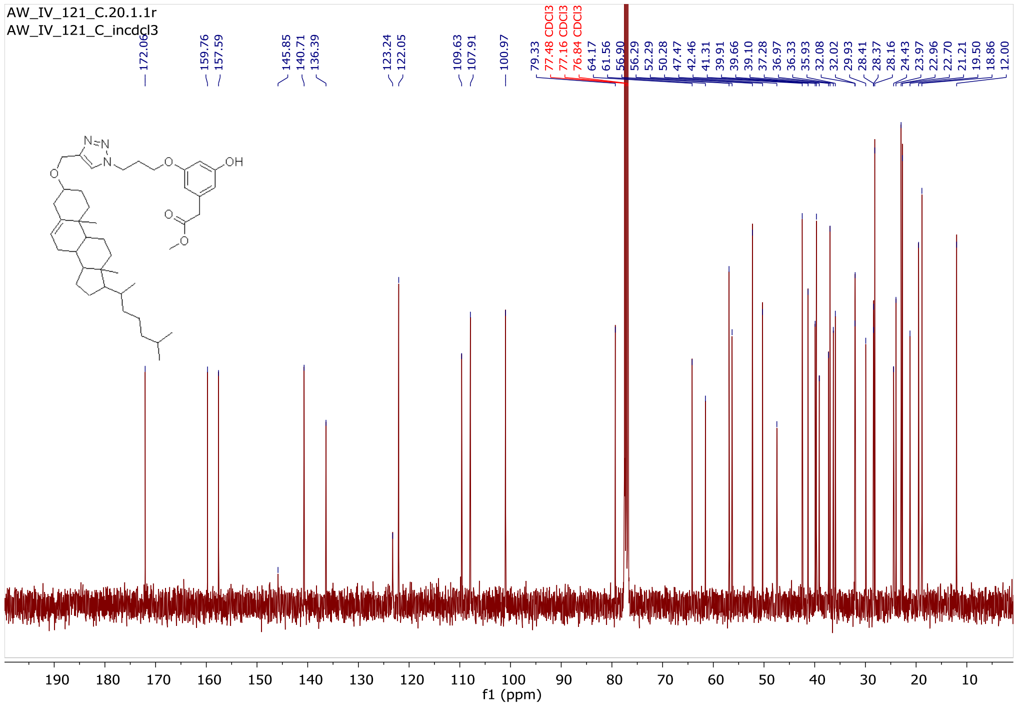

Figure 4. ^{13}C NMR spectrum of **4** in $CDCl_3$.

ATR-IR: 2933, 2866, 1712, 1597 cm^{-1}

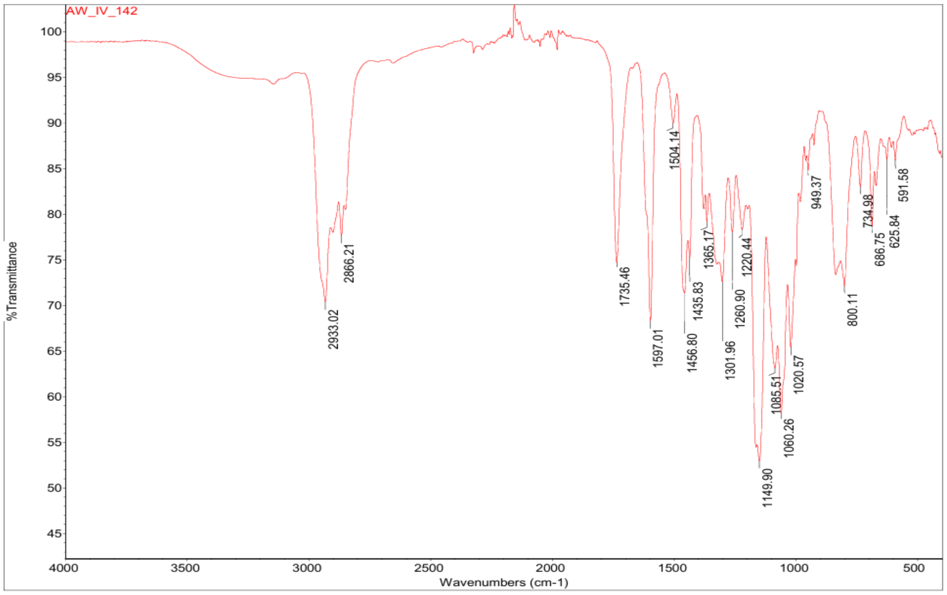

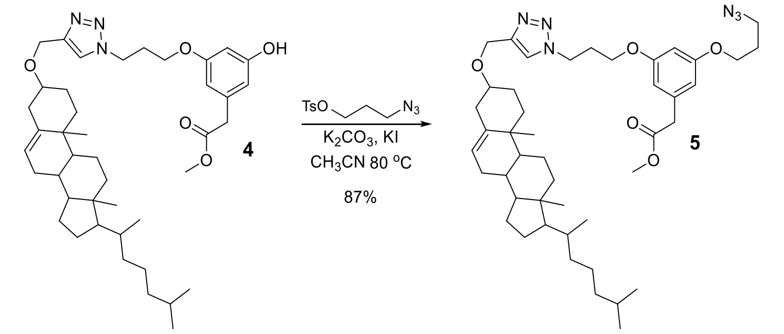

Compound 5. To a solution of compound **4** (177 mg, 0.26 mmol) in CH_3CN (5 mL) were added tosylated azidopropane (66 mg, 0.26 mmol), K_2CO_3 (71 mg, 0.5 mmol), and KI (9 mg, 0.2 mmol) and the resulting solution stirred at 80 °C for 18 h. Satd. aq. $NaHCO_3$ (20 mL) was added, followed by extraction with CH_2Cl_2 (3 × 20 mL). The combined organic phases were washed with brine (20 mL), dried over Na_2SO_4 and the solvent was removed under reduced pressure. The residue was purified by flash-column chromatography using gradient elution (MeOH:CH_2Cl_2 0.1:99.1 to 5:95) to yield compound **5** (173 mg, 0.22 mmol, 87% yield) as a white solid.

TLC (Silica gel, *p*-anisaldehyde, EtOAc:pet. ether 50:50), R_f (**5**) = 0.3

ESI-HRMS (m/z): calcd. for $C_{45}H_{68}N_6O_5$ $[M+Na]^+$ 772.5251, measured 772.5105

^{1}H NMR (400 MHz, $CDCl_3$) δ 7.54 (s, 1H), 6.43 (dt, *J* = 10.5, 1.6 Hz, 2H), 6.35 (t, *J* = 2.3 Hz, 1H), 5.35 – 5.33 (m, 1H), 4.68 (s, 2H), 4.55 (t, *J* = 6.9 Hz, 2H), 4.02 (t, *J* = 5.9 Hz, 2H), 3.96 (t, *J* = 5.7 Hz, 2H), 3.70 (s, 3H), 3.55 (s, 2H), 3.51 (t, *J* = 6.6 Hz, 2H), 3.36-3.28 (m, 1H), 2.43-2.35 (m, 3H), 2.27-2.20 (m, 1H), 2.07-2.02 (m, 2H), 2.00 – 1.92 (m, 2H), 1.88-1.78 (m, 2H), 1.54 (s, 3H), 1.52 – 1.43 (m, 4H), 1.40 – 1.30 (m, 4H), 1.25 (m, 3H), 1.19 – 1.03 (m, 7H), 1.00 (s, 3H), 0.91 (d, *J* = 6.5 Hz, 4H), 0.86 (dd, *J* = 6.6, 1.8 Hz, 6H), 0.67 (s, 3H) ppm.

^{13}C NMR (101 MHz, $CDCl_3$) δ 171.84, 160.09, 159.80, 146.16, 140.84, 136.37, 122.85, 121.97, 108.41, 108.27, 100.33, 79.10, 64.73, 64.24, 61.79, 56.91, 56.30, 52.28, 50.30, 48.36, 47.18, 42.47, 41.49, 39.93, 39.67, 39.18, 37.32, 37.00, 36.34, 35.93, 32.09, 32.04, 30.07, 28.89, 28.46, 28.38, 28.17, 24.44, 23.97, 22.97, 22.71, 21.22, 19.52, 18.87, 12.01 ppm.

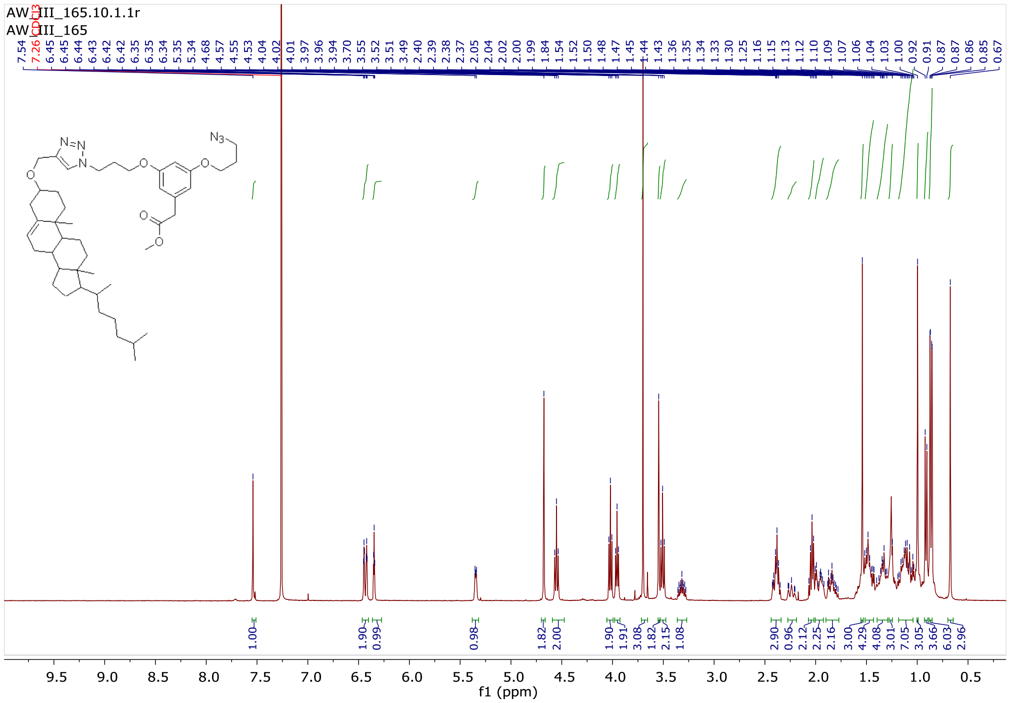

Figure 5. ^{1}H NMR spectrum of **5** in $CDCl_3$.

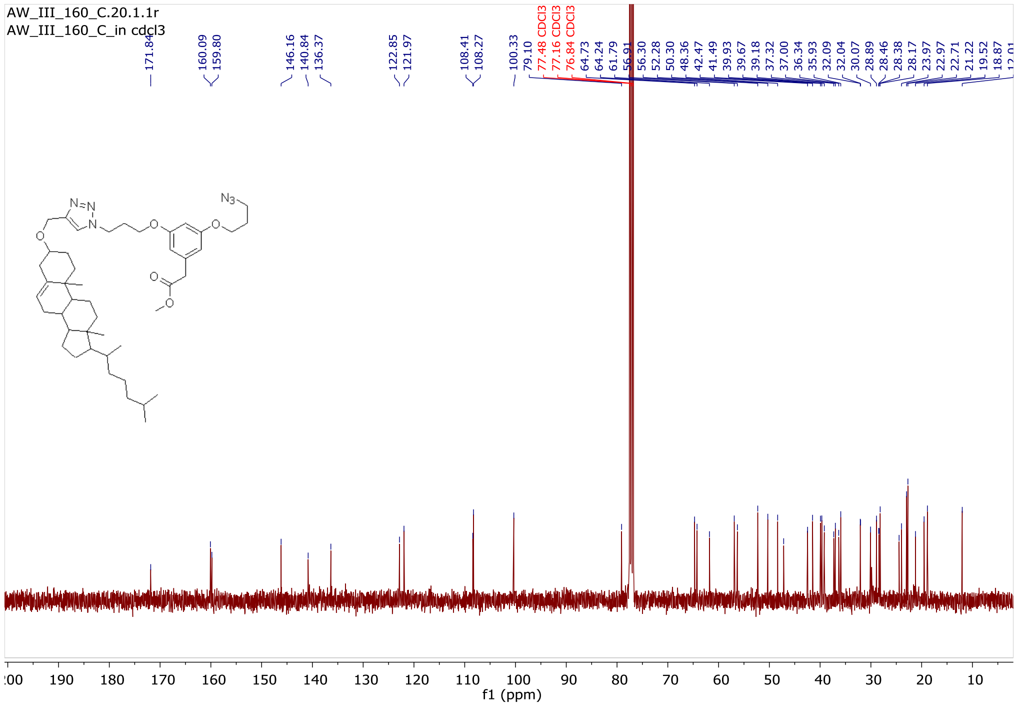

Figure 6. ^{13}C NMR spectrum of **5** in $CDCl_3$.

ATR-IR: 2930, 2093, 1735, 1594 cm^{-1}

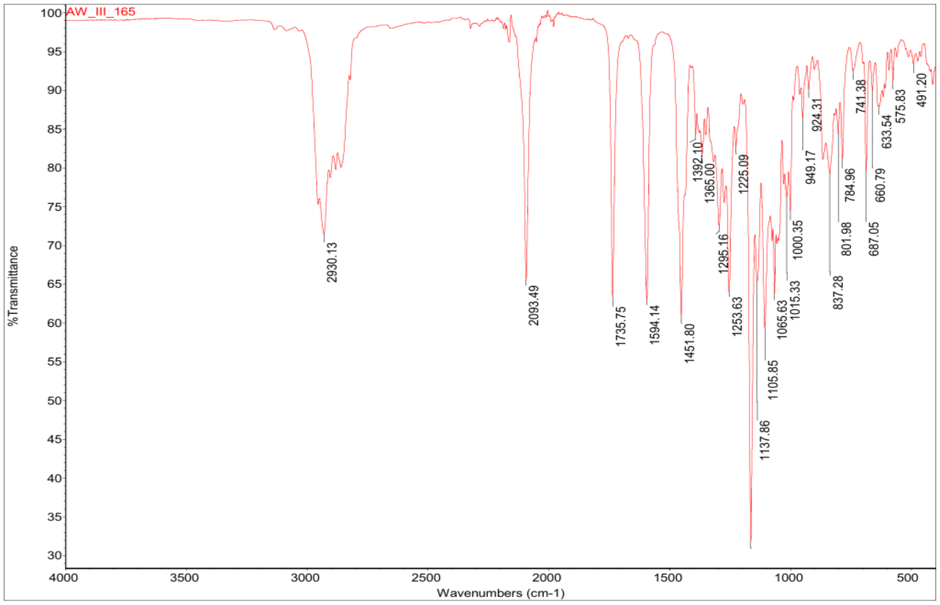

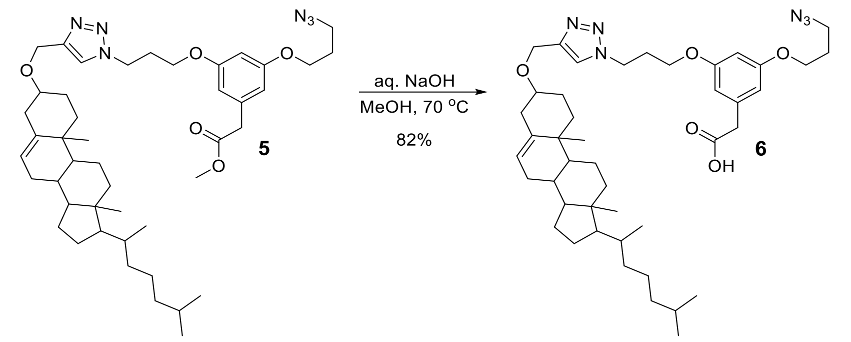

Compound 6. To a solution of compound **5** (50 mg, 0.12 mmol) in MeOH (20 mL) was added an aq. NaOH solution (5 mL, 2.5 M) and the resulting solution stirred at 70 °C for 24 h. The solvent was removed under reduced pressure and aqueous HCl (30 mL, 1 M) solution was added, followed by extraction with CH_2Cl_2 (3 × 20 mL). The combined organic phases were washed with brine (20 mL), dried over Na_2SO_4 and the solvent was removed under reduced pressure. The residue was purified by thin-layer chromatography using an isocratic elution (MeOH:CH_2Cl_2 5:95) to yield compound **6** (40 mg, 0.05 mmol, 82% yield) as a white solid.

TLC (Silica gel, *p*-anisaldehyde, MeOH:CH_2Cl_2 0.5:9.5), R_f (**6**) = 0.3

ESI-HRMS (m/z): calcd. for $C_{44}H_{66}N_6O_5$ $[M+Na]^+$ 566.2949, measured 566.290

^{1}H NMR (400 MHz, $CDCl_3$) δ 7.52 (s, 1H), 6.40 (d, *J* = 9.8 Hz, 2H), 6.30 (s, 1H), 5.34 (d, *J* = 5.0 Hz, 1H), 4.65 (s, 2H), 4.49 (t, *J* = 6.3 Hz, 2H), 3.96 (t, *J* = 5.7 Hz, 2H), 3.90 (t, *J* = 5.9 Hz, 2H), 3.46 (t, *J* = 6.6 Hz, 4H), 3.36 – 3.29 (m, 1H), 2.41-2.38 (m, 1H), 2.32 – 2.21 (m, 3H), 2.02-1.92 (m, 5H), 1.87 – 1.78 (m, 2H), 1.58 – 1.42 (m, 7H), 1.35 – 1.30 (m, 3H), 1.25 (s, 3H), 1.16 – 1.03 (m, 7H), 0.99 (s, 3H), 0.91 (d, *J* = 6.5 Hz, 4H), 0.86 (dd, *J* = 6.6, 1.8 Hz, 6H), 0.67 (s, 3H) ppm.

^{13}C NMR (101 MHz, $CDCl_3$) δ 175.88, 160.06, 159.77, 145.98, 140.72, 136.25, 122.99, 122.00, 108.59, 108.23, 100.47, 79.21, 64.71, 64.25, 61.61, 56.88, 56.28, 50.27, 48.33, 47.21, 42.45, 41.58, 39.90, 39.65, 39.09, 37.27, 36.96, 36.32, 35.92, 32.07, 32.01, 30.00, 28.86, 28.39, 28.36, 28.14, 24.42, 23.96, 22.95, 22.70, 21.20, 19.49, 18.85, 11.99 ppm.

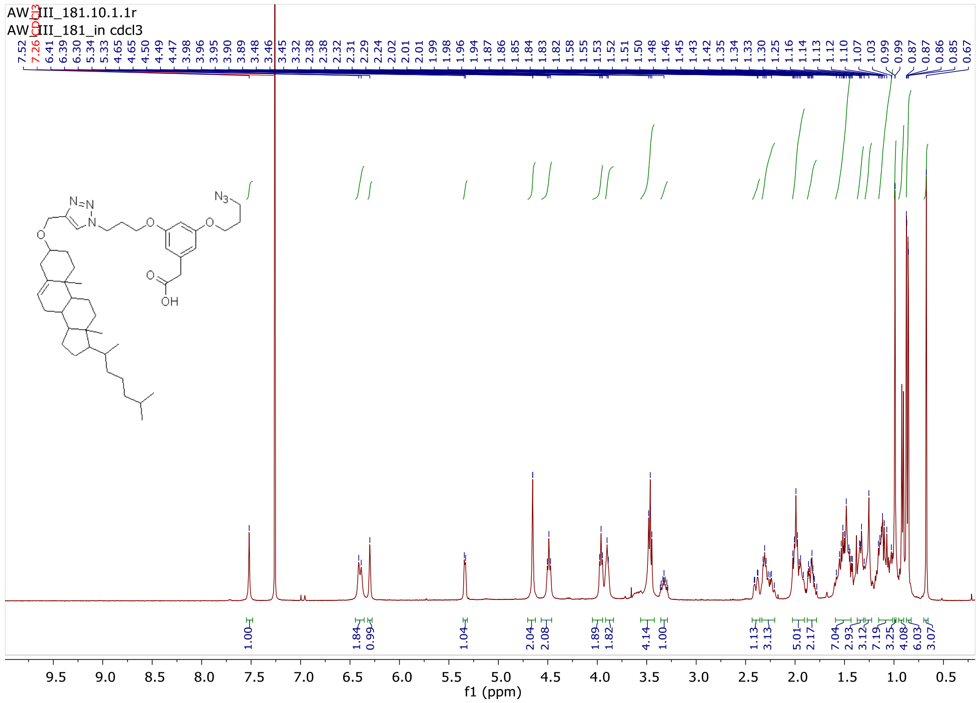

Figure 7. ^{1}H NMR spectrum of **6** in $CDCl_3$.

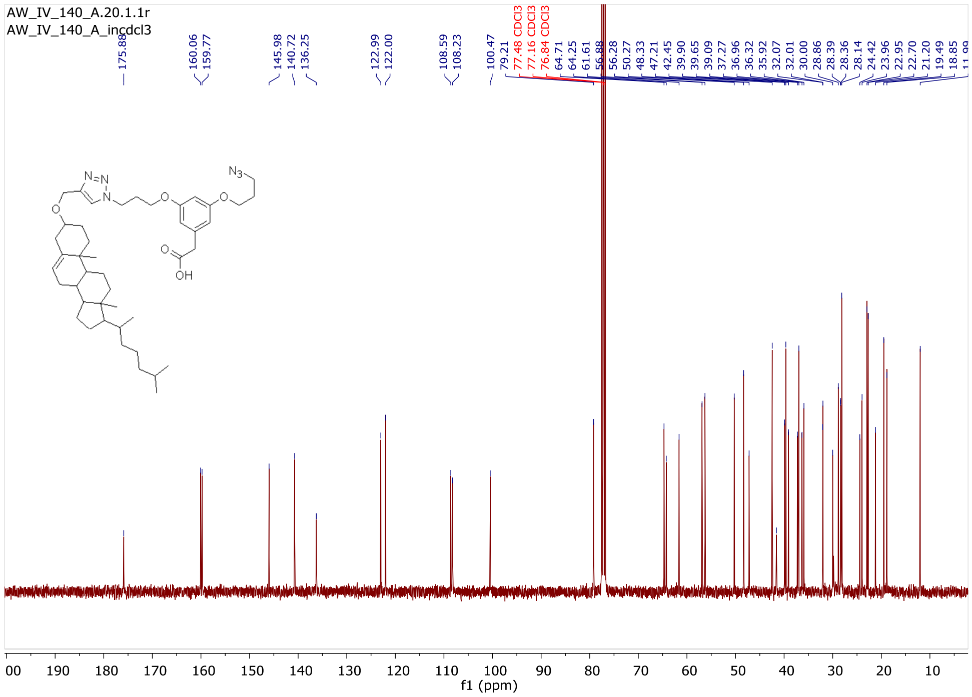

Figure 8. ^{13}C NMR spectrum of **6** in $CDCl_3$.

ATR-IR: 2931, 2089, 1717, 1595 cm^{-1}

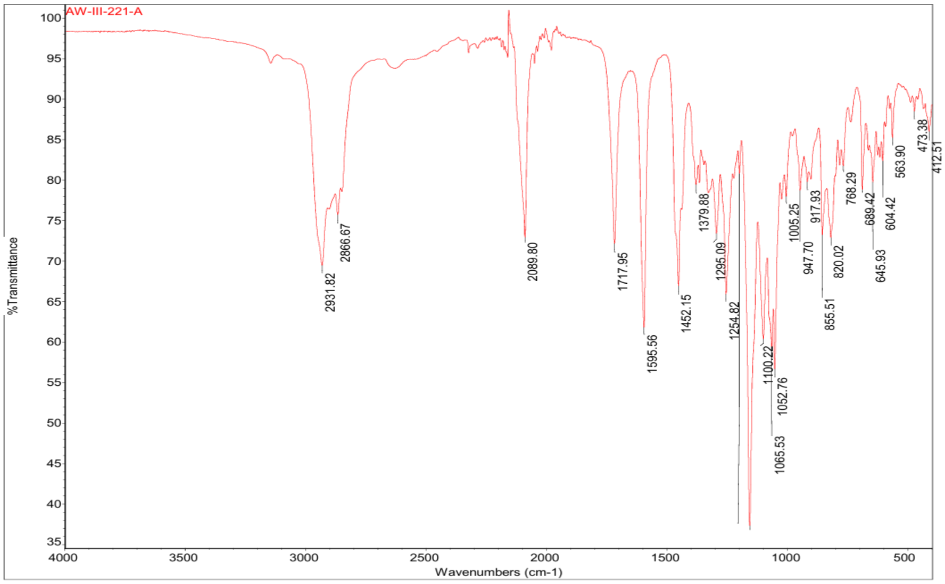

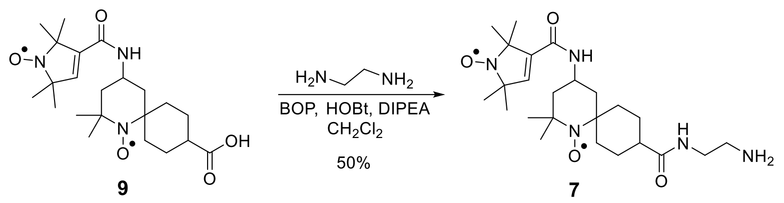

Compound 7. To a stirred solution of compound **9**[3] (18 mg, 0.043 mmol) in CH_2Cl_2 (0.4 mL) were added BOP (28.3 mg, 0.06 mmol), HOBt (8.6 mg, 0.06 mmol), DIPEA (0.022 mL, 0.13 mmol) at 22 °C, followed by ethylene diamine (0.114 mL, 1.71 mmol). The reaction mixture was stirred for 3 h, aq. NaOH solution (20 mL, 2.5 M) was added, and the reaction mixture was extracted with CH_2Cl_2 (3 × 10 mL). The combined organic phases were washed with brine (10 mL), dried over Na_2SO_4 and the solvent was removed under reduced pressure. The residue was purified by prep-TLC (MeOH:CH_2Cl_2 10:90) to yield compound **7** (20 mg, 0.04 mmol, 50% yield) as an orange liquid. The purity of radical **7** was ascertained on Macherey-Nagel Nucleodur C18 Pyramid 4.6 × 150 mm analytical HPLC column with UV detection at λ = 254 nm using gradient A.

<u>TLC</u> (Silica gel, UV, MeOH:CH_2Cl_2 1:9), R_f (**7**) = 0.1

<u>ESI-HRMS (m/z)</u>: calcd. for $C_{24}H_{41}N_5O_4$ $[M+H]^+$ 464.3231, measured 464.3154

<u>EPR</u> (MeOH):

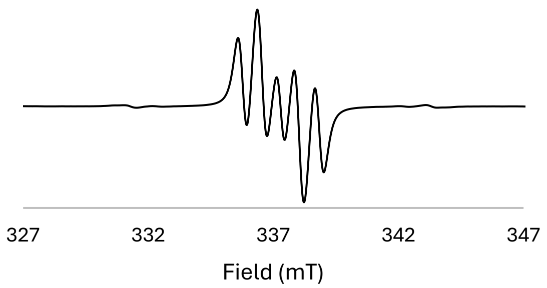

<u>HPLC</u>:

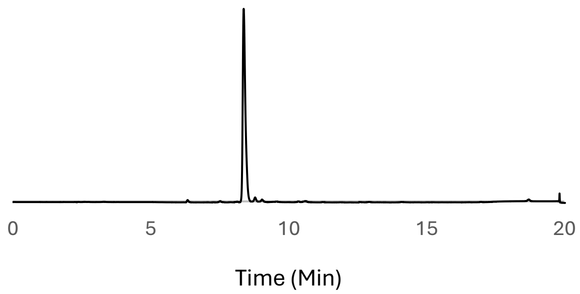

ATR-IR: 3305, 2926, 1651 cm^{-1}

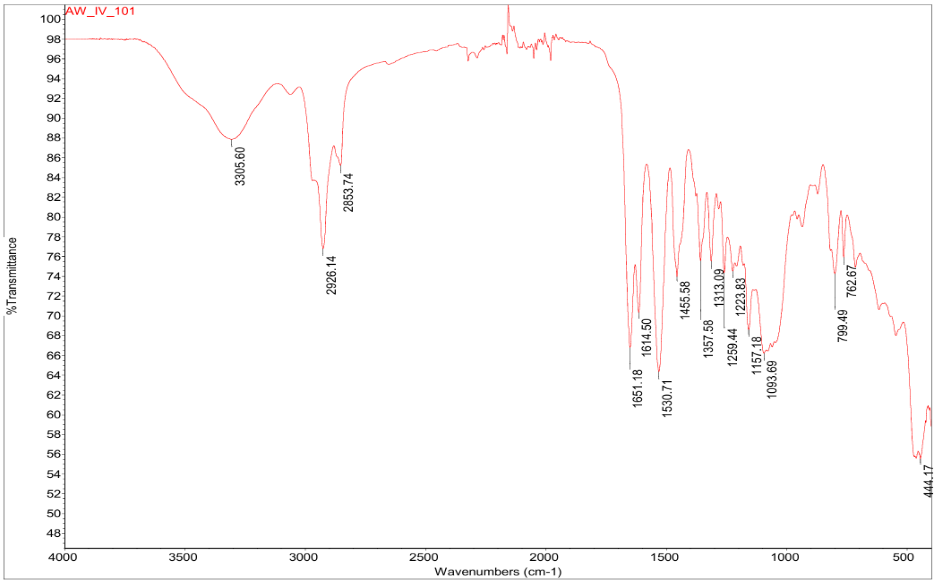

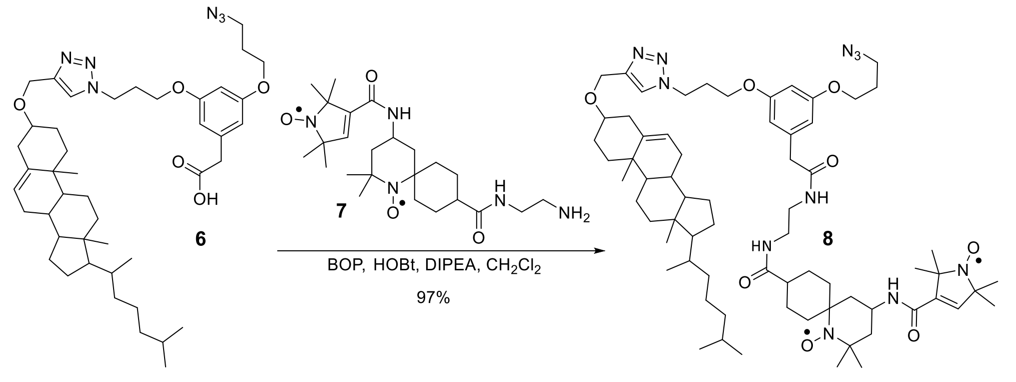

Compound 8. To a stirred solution of compound **6** (15 mg, 0.02 mmol) in CH_2Cl_2 (0.4 mL) were added BOP (13 mg, 0.03 mmol), HOBt (4 mg, 0.03 mmol), DIPEA (0.01 mL, 0.06 mmol) at 22 °C, followed by **7** (10 mg, 0.02 mmol). The reaction mixture was stirred for 6.5 h, satd. $NaHCO_3$ solution (10 mL) was added, and the reaction mixture was extracted with CH_2Cl_2 (3 × 10 mL). The combined organic phases were dried over Na_2SO_4 and the solvent was removed under reduced pressure. The residue was purified by prep-TLC (MeOH:CH_2Cl_2 5:95) to yield **8** (25 mg, 0.02 mmol, 68% yield) as an orange solid. The purity of radical **8** was ascertained on Pursuit 5 C8 Pyramid 4.6 × 250 mm analytical HPLC column with UV detection at λ = 254 nm using gradient B.

TLC (Silica gel, *p*-anisaldehyde, MeOH:CH_2Cl_2 0.5:9.5), R_f (**8**) = 0.3

ESI-HRMS (m/z): calcd. for $C_{68}H_{105}N_{11}O_8$ $[M+Na]^+$ 1226.8040, measured 1226.7916

EPR (MeOH):

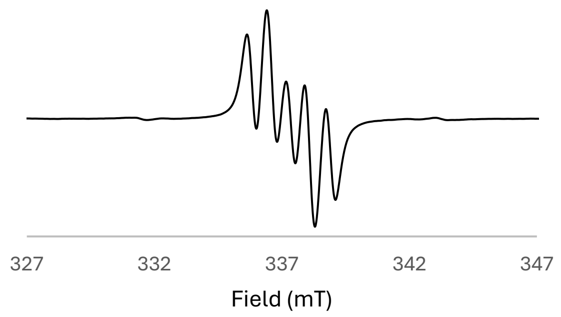

HPLC:

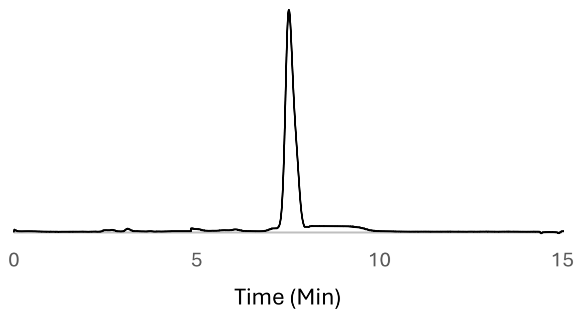

ATR-IR: 2930, 2096, 1659, 1595 cm^{-1}

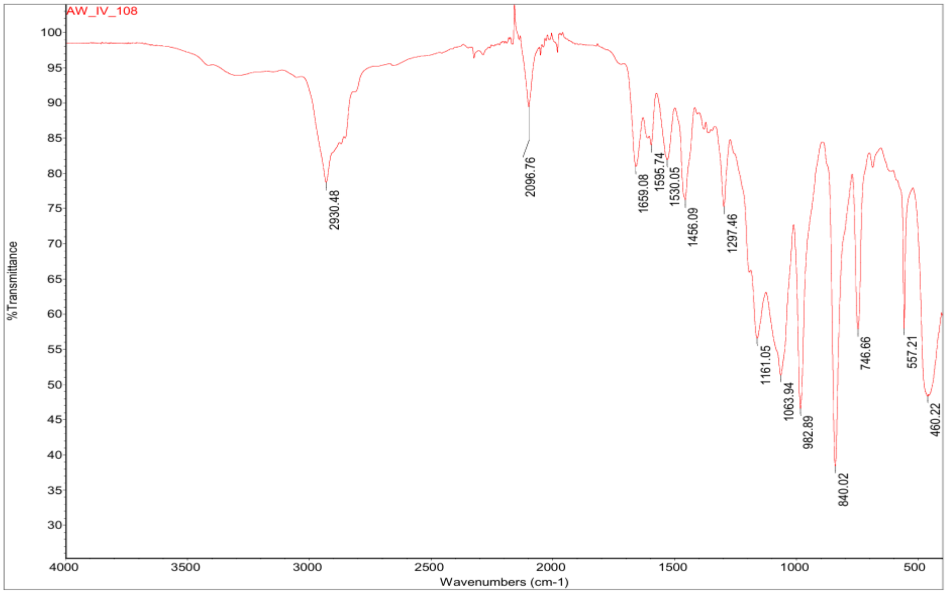

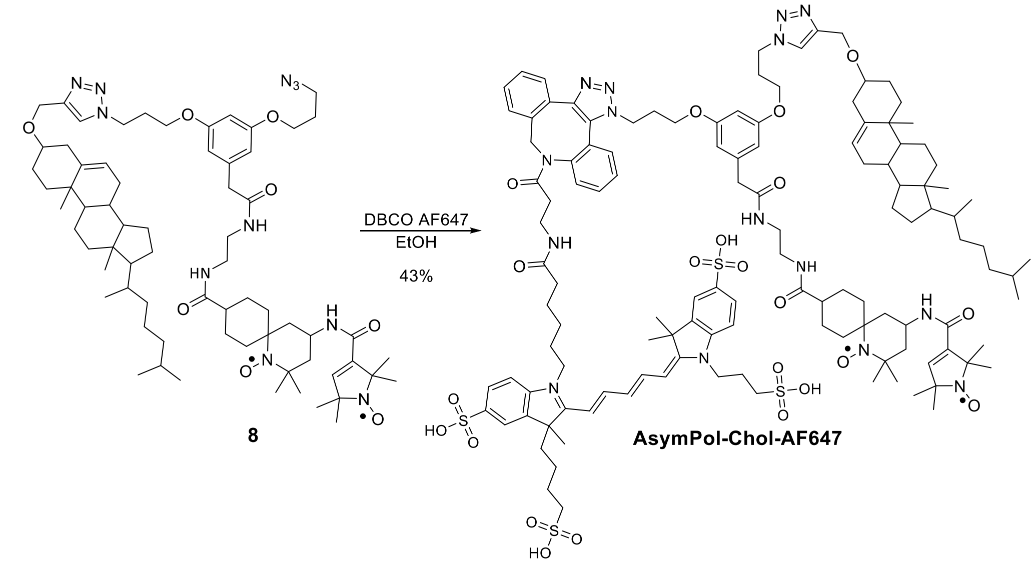

AsymPol-Chol-AF647. To a solution of compound **8** (2 mg, 1.7 µmol) in EtOH (8 µL) was added a solution of DBCO AF647 (1mg, 0.89 µmol) in EtOH (8 µL) at 22 °C. The reaction mixture was diluted with EtOH (500 µL) after 1 h and purified by Pursuit 5 C18 Pyramid 4.6 × 250 mm analytical column with UV detection at λ = 254 and 663 nm, using gradient C to yield AsymPol-Chol-AF647 (0.9 mg, 0.39 µmol, 43% yield) as a blue solid. The purity of AsymPol-Chol-AF647 was ascertained on Macherey-Nagel Nucleodur C18 Pyramid 4.6 × 150 mm analytical HPLC column with UV detection at λ = 254 and 663 nm using the same gradient.

TLC (Silica gel, MeOH:CH_2Cl_2 4:6), R_f (AsymPol-Chol-AF647) = 0.4

ESI-HRMS (m/z): calcd. for $C_{122}H_{165}N_{15}S_4O_{22}$ $[M-3H]^{3-}$ 772.6983, measured 772.6985

EPR (EtOH):

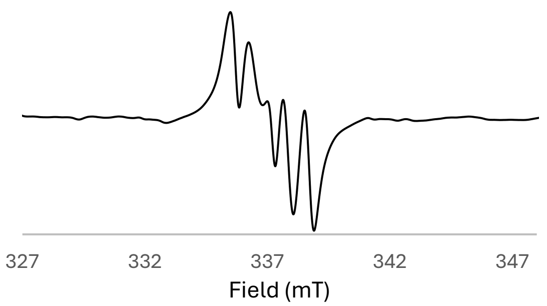

HPLC:

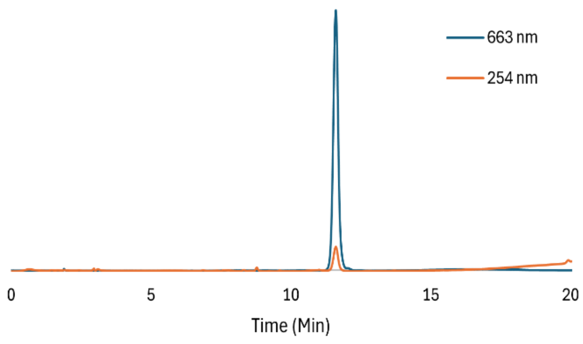

UV-Vis spectrum:

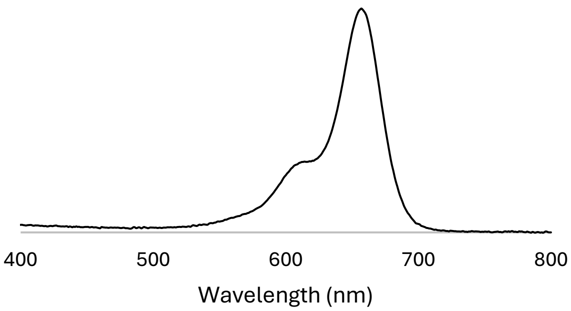

Fluorescence spectrum:

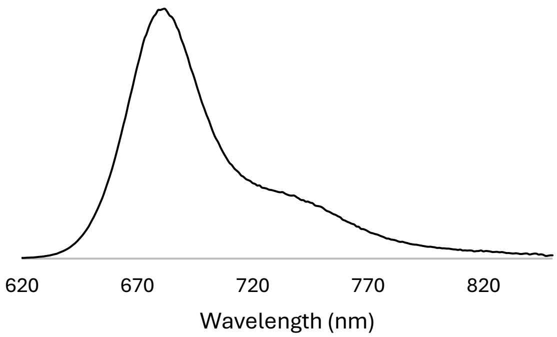

SI-2 Materials and Methods

Cell culture

JLat 9.2 T cells were cultured in RPMI supplemented with 10 % fetal bovine serum, 2 mM L-glutamine, 10 U/mL penicillin and streptomycin and 20 mM sodium pyruvate and grown at 37 °C with 5 % CO_2 in a humidified incubator.

Cell viability

Cell viability was determined with trypan blue staining where an aliquot of JLat 9.2 T cells was diluted ½ with 0.9% trypan blue. Live cells were then counted under a light microscope using a haemocytometer where the total number of cells was counted and the number of blue (dead cells) determined.

Sample preparation

JLat 9.2 T cells were counted and 40 x 10^6 cells were washed with 20 mM Tris with 137 mM NaCl in D_2O. The cells were pelleted, the supernatant removed and tripod-cholesterol dissolved in d_6-DMSO added at the concentrations indicated to the cell pellet along with additional d_6-DMSO to give a final concentration of 10% v/v. The sample was gently mixed and transferred to a 3.2 mm sapphire DNP rotor by centrifugation at 1500 rpm. 1 µL of prepared cells (~1 x 10^6 cells) were then taken and resuspended in 500 µL phosphate buffered saline (PBS). The remaining cells in the rotor were flash frozen in liquid nitrogen and stored in liquid nitrogen until DNP analysis. Of the 500 µL cell suspension taken, an aliquot was then taken for trypan blue analysis, the rest was taken immediately for live cell imaging by confocal microscopy.

Solid-state DNP-NMR

DNP solid-state experiments were acquired on a 400 MHz (9.4 T) wide bore Bruker spectrometer equipped with an HXY low temperature probe at 86 K microwaves off and 91 K microwaves on. Microwaves were provided by a mounted Klystron with 5.1 W output and 9 kHz MAS. Cross polarization was achieved using a 70% linear ramp with ^{1}H spin locking frequency of 59 kHz and ^{13}C spin lock at 50 kHz. T_B curves were generated with saturation recovery experiments with saturation pulses trains on ^{1}H and ^{13}C followed by echo detection and 96 kHz SPINAL64 decoupling over acquisition and 128 scans. All experiments were acquired with a recycle delay of 10 s (optimization shown in **SI-11**)

Data was processed in topspin 4.4.0. T_B buildup curves were determined by plotting peak intensity as a function of polarization delay and fitting to equation 1 in GraphPad Prism.

$$I(t) = I_0\left(1 - e^{t/T_1}\right) \quad (1)$$

Enhancements were determined as the ratio of the signal intensity with (I_{ON}) and without (I_{OFF}) microwave irradiation:

$$\varepsilon = \frac{I_{ON}}{I_{OFF}} \quad (2)$$

Error bars in the enhancement buildups were determined by error propagation over the microwave on and microwave off signal to noise:

$$\sigma_z = z\sqrt{\frac{\sigma_x^2}{x} + \frac{\sigma_y^2}{y}} \quad (3)$$

Confocal microscopy

2.3 × 10^6 JLat 9.2 T cells were washed with phosphate buffered saline (PBS) and incubated for 15 min in PBS supplemented with a 1:100 rabbit anti-GM1 antibody and a 1:1000 Anti-Rabbit IgG CF 568 antibody (Sigma-Aldrich). The cells were washed twice with 1 mL PBS and supplemented with 10% DMSO and transferred to 8 well glass slides (Ibidi). Live cell confocal microscopy was performed with a Nikon NSTORM (Nikon UK, Ltd) system equipped with a Re-scan Confocal Microscope RCM1 (Confocal.nl, Amsterdam, the Netherlands). We used an sCMOS camera (Orca Flash 4.0 V2) and a Nikon SR Apochromat TIRF objective 100x / 1.49 with oil immersion. The different laser excitations were at 561 nm, and 647 nm. The setup was fully controlled, and image acquisition was performed using the NIS-Elements software (Nikon). The implemented re-scan unit provides an enhancement in resolution from 240 nm to 170 nm. Images were processed in ImageJ where the brightness was adjusted such that the brightest pixels had a greyscale value of 50 and the contrast adjusted to make fluorescent structures distinguishable from non-fluorescent structures.

Colocalization analysis

Colocalization analysis was performed using Coloc 2 plugin as well as binary overlap method on images of 0.19 nmol/million PA loaded JLat 9.2 T cells. Images of AF647 (PA) and AF561 (GM1) were background subtracted after automatic brightness and contrasting. Colocalization of background subtracted images was quantified using Coloc 2 plugin of ImageJ on using Costes method of automatic thresholding. Colocalization was measured by the Manders coefficient is given as a measure of GM1-positive pixels that colocalize with PA+ pixels according to:

$$M2 = \frac{\sum I_2(pixels\ where\ I_1 > 0)}{\sum I_2(all\ pixels)}$$

The Pearson's correlation was calculated by:

$$r = \frac{\sum(I_1 - \bar{I}_1)\,(I_2 - \bar{I}_2)}{\sqrt{\sum(I_1 - \bar{I}_1)^2\,(I_2 - \bar{I}_2)^2}}$$

where $\bar{I}_1$ is the mean intensity of channel 1 and $\bar{I}_2$ is the mean intensity of channel 2 as a measure of intensity correlation between AF647 intensity and AF561 intensity. Colocalization was also determined using binary masks on background subtracted images manually thresholded. Fluorescence intensity thresholds were determined manually in ImageJ by visual inspection using the Fire (heatmap) lookup table (LUT) to identify the transition between background and true signal. The same thresholding criteria were applied consistently across all images within each experimental condition. Thresholded images were converted to binary masks and the ratio of pixel overlap between the AF647 and AF561 channels was determined.

Simulations

Simulations of polarization buildup were performed using the MATLAB code published by Pinon A *et al*.[4] and MATLAB was accessed through NMRBox[5]. Best fit simulated buildups to the experimental data were performed using reduced χ^2 analysis. Each experimental point was weighted according to the variance of the propagated error. The data was fit using bounded least-squares method to minimize the reduced χ^2 value to 1 of the fits of the enhancement buildup, the polarization buildup with microwaves and the polarization buildup without microwaves.

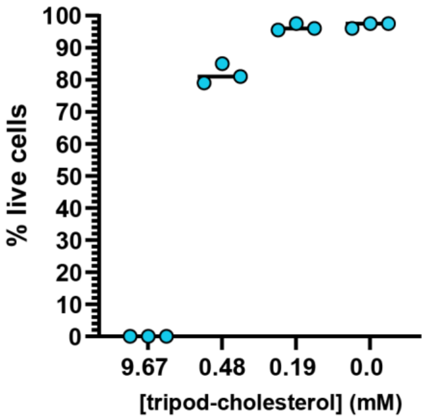

SI-3. *Cell survival after loading with bimodal-cholesterol.* Live cells were loaded with the indicated concentrations of bimodal-cholesterol in preparation for DNP. Prior to flash freezing, 2 μL of cells were taken for trypan blue counting. The horizontal bar shows the mean of triplicate counts from each sample.

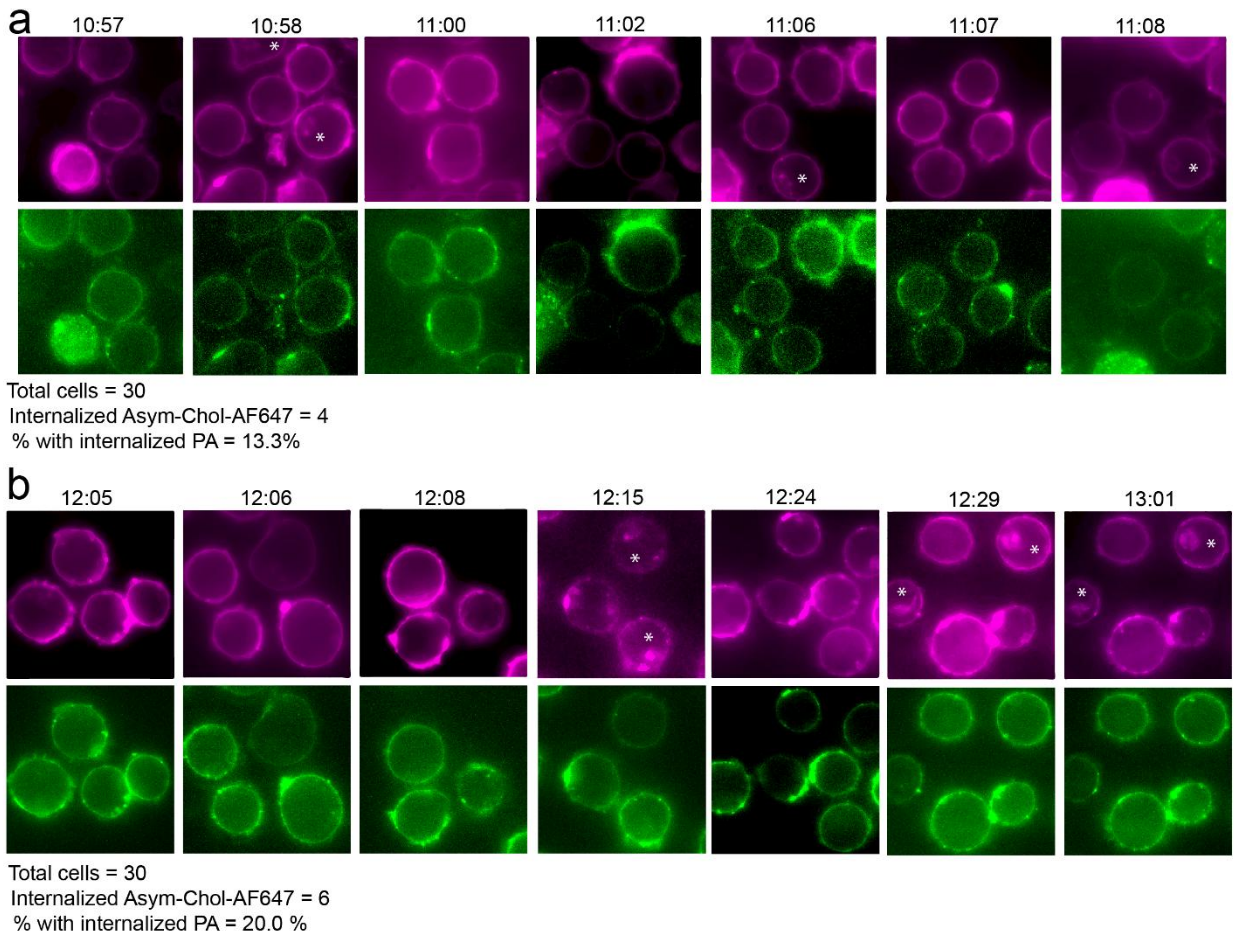

SI-4. *Time course of Asym-Chol-AF647 internalization*. a) JLat 9.2 T cells loaded with 0.48 nmol/million Asym-Chol-AF647. b) JLat 9.2 t cells loaded with 0.19 nmol/million Asym-Chol-AF647. Images at 35, 45, 50 and 60 min are equatorial plane images from a z-stack series. * indicates cells with AF647 internalization. The times given are the total time cells were incubated with the PA at the time of imaging. Staining with Asym-Chol-AF647 is shown in magenta and staining with GM1 is shown in green.

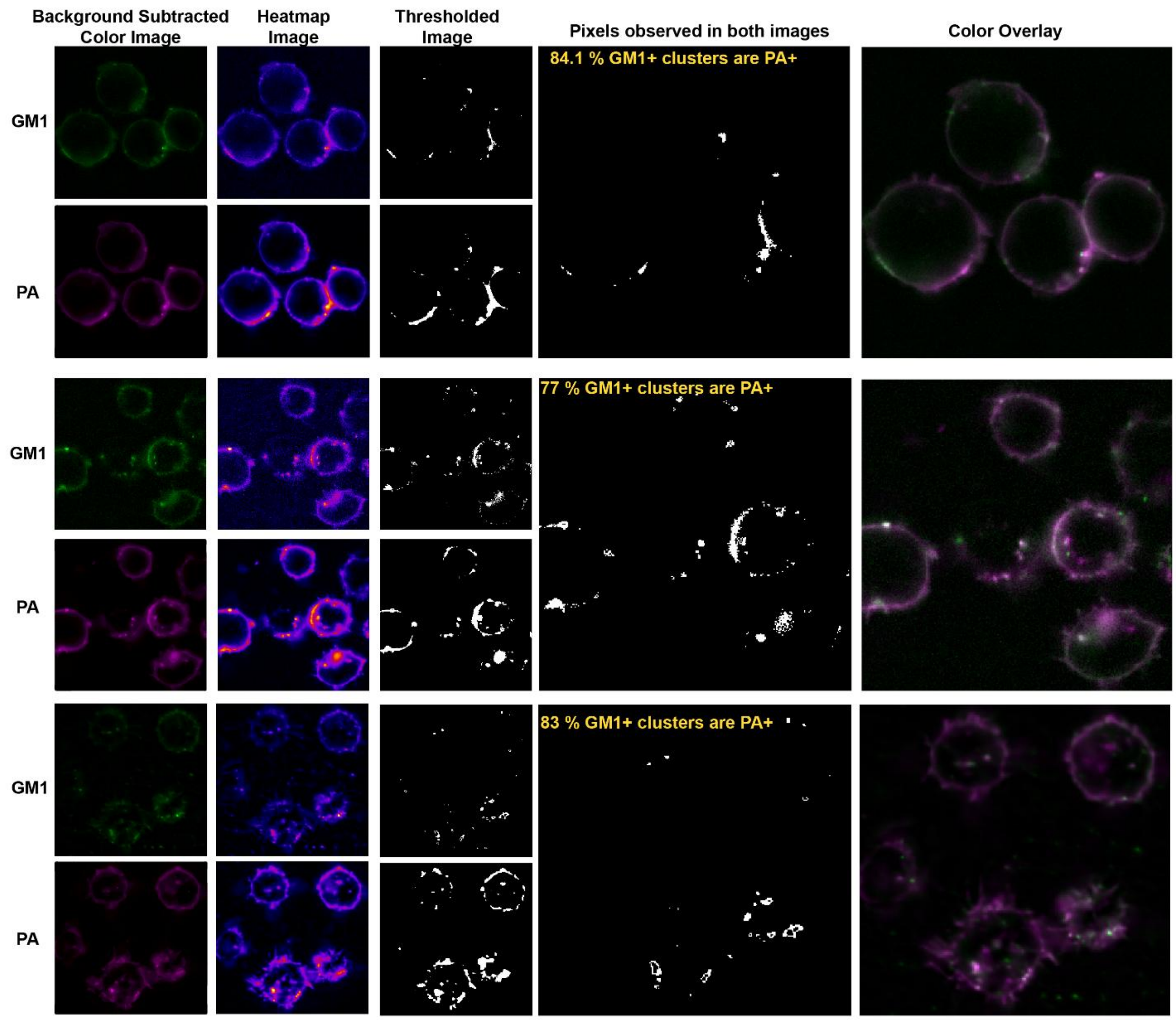

SI-5: *Colocalization analysis by binary overlap of JLat 9.2 T cells loaded with 0.19 nmol/million Asym-Chol-AF647 (PA).* Raw confocal images were brightened automatically and then the background subtracted. The images were then converted to heatmaps for manual thresholding in which the threshold was set to areas of red intensity increasing to white to create binary masks used to determine the pixel overlap from both images. The overlapped pixels are then counted and expressed as the ratio of overlapping pixels with the number of GM1+ pixels to obtain the GM1+ colocalization coefficient. For comparison, the color overlay is shown. White intensity indicates overlapping pixels. The processing for 3 of the 5 analyzed images are shown.

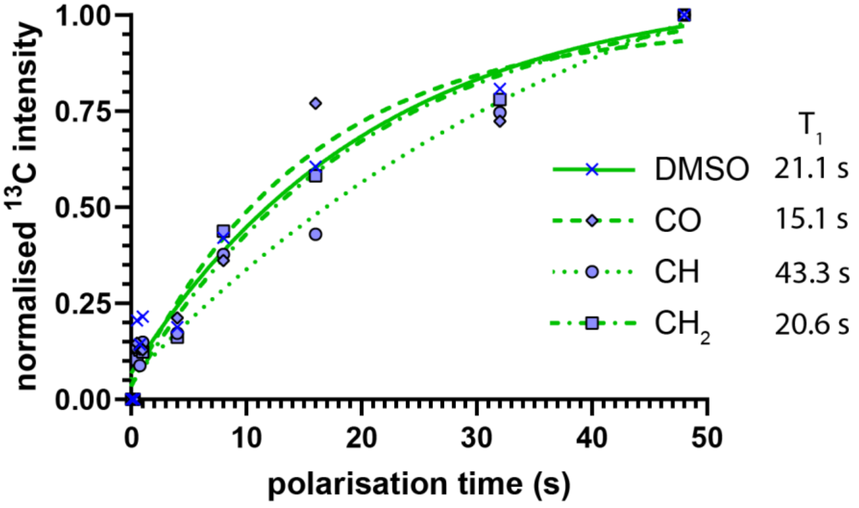

SI-6. *T_1 buildup for JLat 9.2 T cells in the absence of radical.* Saturation recovery experiment of JLat 9.2 T cells cryopreserved with 10% d_6-DMSO. The normalized signal intensity represents the peak intensity of each chemical group indicated. Data was acquired at 600 MHz ^{1}H Larmor frequency at 9 kHz.

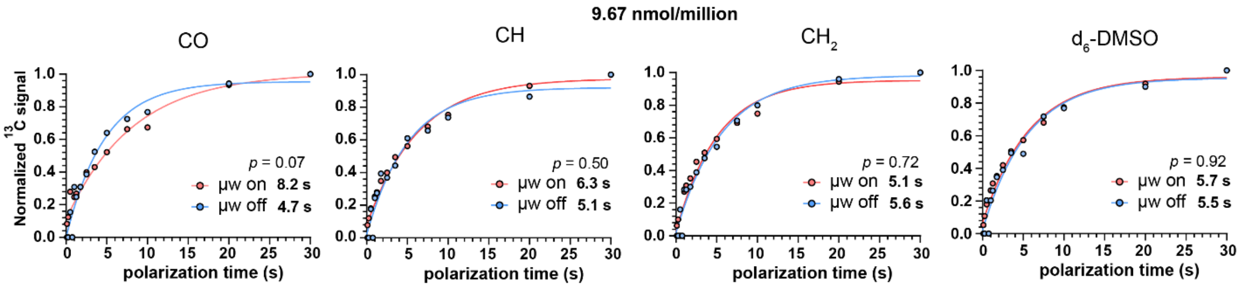

SI-7. *Simulated T_B fit to experimental polarization buildup data in 9.67 nmol/million PA loaded cells*. The data was fit to the exponential growth curve described in the methods, within GraphPad Prism. The significance of the difference between microwave on (red curves) and microwave off (blue curves) was determined using an F-test of the difference in 95% confidence intervals of the fitted rate constant $1/T_B$. Differences were considered significant when $p \leq 0.05$.

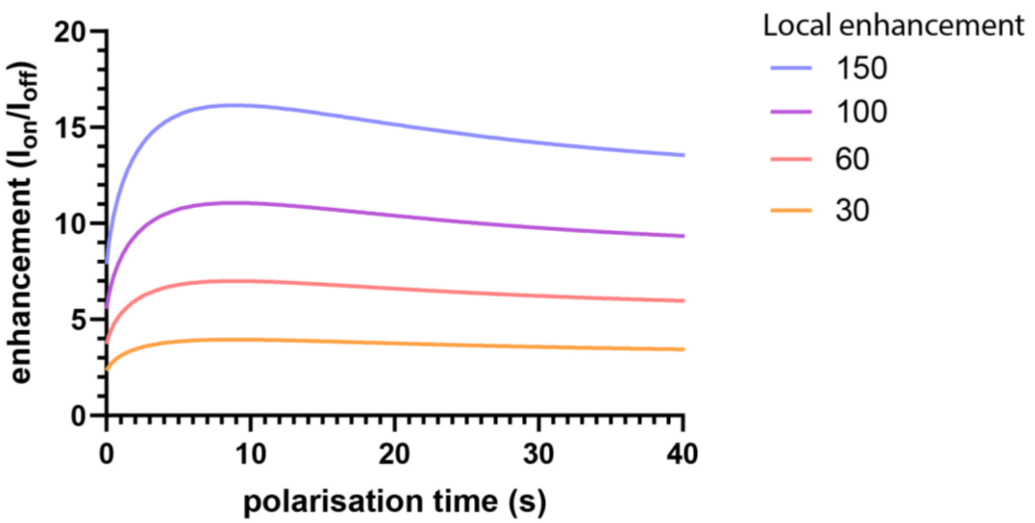

SI-8. *Increasing the local enhancement scales the global enhancements when the local diffusion coefficient is smaller than the bulk diffusion coefficient*. Simulations are executed with MATLAB code as described in the experimental methods section using the following parameters: $L_{uncoupled}$ = 5 µm, $L_{coupled}$ = 6 nm, $T_{1,coupled}$ = 5.0 s, $T_{1,uncoupled}$ = 25 s, $D_{coupled}$ = 0.00000215 µm^2.s^{-1}, ($D_{uncoupled}$) = 0.001 µm^2.s^{-1}, depolarization = 0.78, and θ = 0.99.

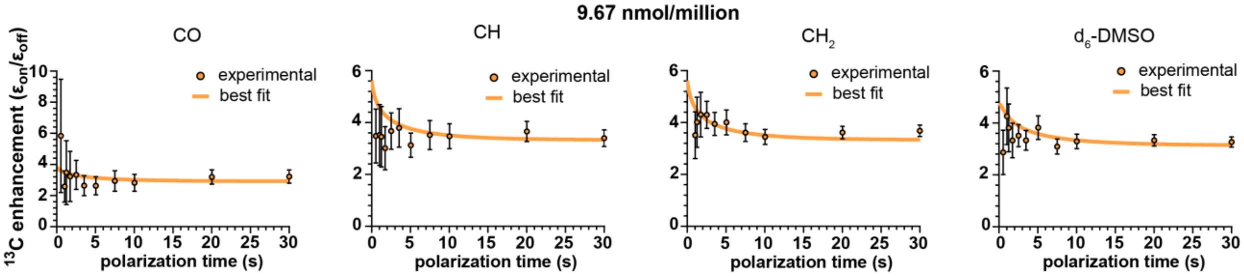

SI-9. *Enhancement buildup curves of JLat 9.2 T cells loaded with 9.67 nmol/million AsymPol-Chol-AF647.* ^{13}C enhancements are calculated as the ratio of the intensity of the indicated ^{13}C signals with (I_{ON}) and without (I_{OFF}) microwave irradiation as a function of time. Error bars indicate the propagated error of the peak intensity. Experimental data was fit (orange line) using the MATLAB code published in Pinon. A. *et al.* [4] using the following model parameters: $T_{1,uncopled}$ = 25.0 s, spin diffusion coefficient of the uncoupled nuclei ($D_{uncoupled}$) = 0.001 µm^2.s^{-1}, $L_{uncoupled}$ = 5.0 µm, $L_{coupled}$ = 6 nm, L_{border} between hyperfine coupled nuclei and uncoupled nuclei spins = 2 nm.

Table S1. Best Fit parameters for ^{13}C enhancement buildups. The best fit parameters were determined by reduced χ^2 analysis of the enhancement buildup data and fit to a model system in which the $L_{uncoupled}$ = 5 μm, $L_{coupled}$ = 6 nm, $D_{uncoupled}$ = 0.001 $\mu m^2.s^{-1}$, $T_{1,uncoupled}$ = 25.0 s, depolarization = 0.78, and θ = 0.99.

**0.19 nmol/million**	**d_6-DMSO**	**CO**	**CH**	**CH_2**
$T_{B,coupled}$ (s)	9.4	7.6	11.2	7.7
ε_{local}	161	146	183	141
$D_{coupled}$ ($\mu m^2.s^{-1}$)	2.15 x 10^{-6}	2.15 x 10^{-6}	2.15 x 10^{-6}	2.15 x 10^{-6}
**0.48 nmol/million**	**d_6-DMSO**	**CO**	**CH**	**CH_2**
$T_{B,coupled}$ (s)	1.5	2.5	3.2	1.4
ε_{local}	20	55	75	63
$D_{coupled}$ ($\mu m^2.s^{-1}$)	2.15 x 10^{-6}	0.001	0.001	8.7 x 10^{-4}

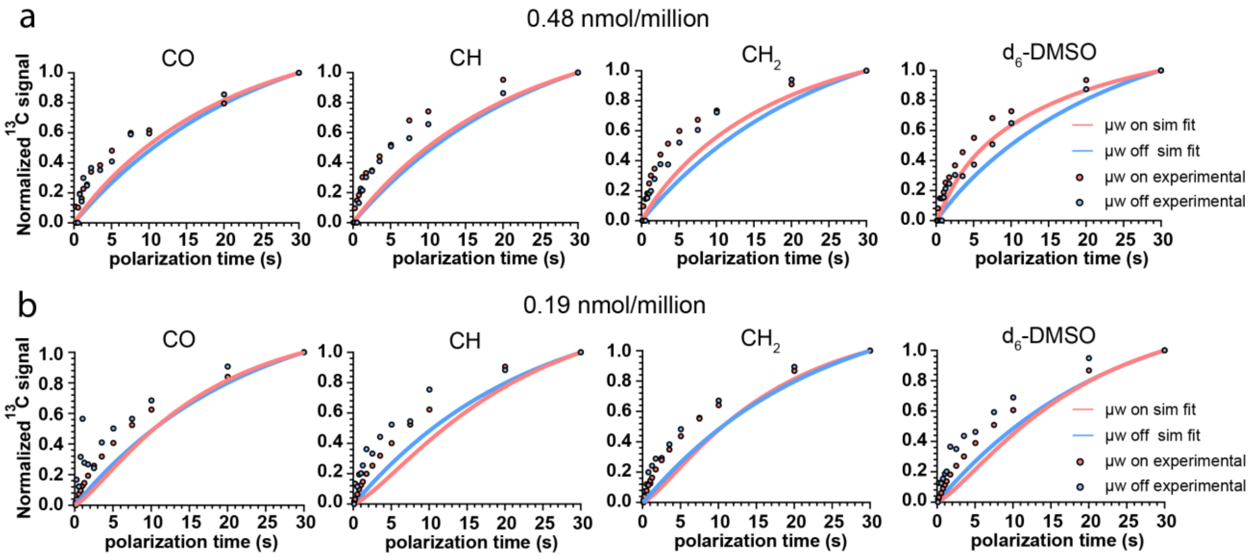

SI-10: *Simulated signal buildup curves using the best fit parameters for the enhancement data shown in* ***Figure 6***. The curves were generated using the following model parameters: $T_{1,uncopled}$ = 25.0 s, spin diffusion coefficient of the uncoupled nuclei ($D_{uncoupled}$) = 0.001 μm^2.s^{-1}, $L_{uncoupled}$ = 5.0 μm, $L_{coupled}$ = 6 nm, L_{border} between hyperfine coupled nuclei and uncoupled nuclei spins = 2 nm. Additional fit parameters are reported in **Table S1** and are the same as those used to fit the enhancement buildups shown in **Figure 6** of the main manuscript.

a

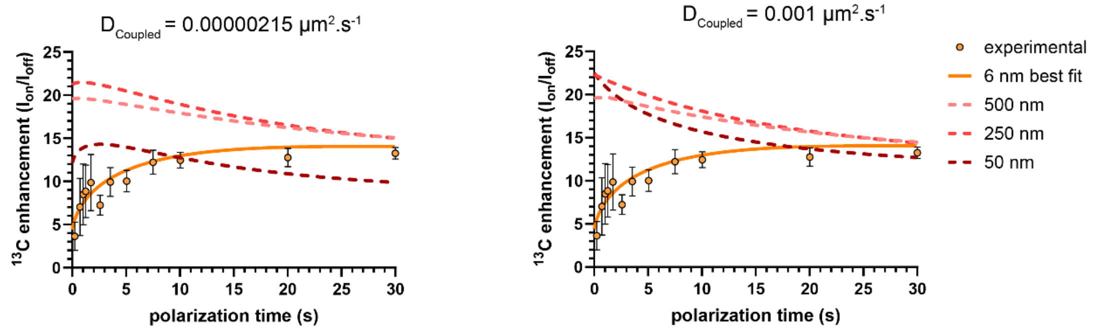

$L_{uncoupled}$	ε_{local}	$T_{B,coupled}$ (s)	$T_{1,uncoupled}$ (s)	$D_{coupled}$ (µm^2.s^{-1})
6 nm	161	9.9	25.0	2.15×10^{-6}
50 nm	119	9.8	25.0	2.15×10^{-6}
250 nm	65	7.1	25.0	2.15×10^{-6}
500 nm	45	9.1	25.0	2.15×10^{-6}

$L_{uncoupled}$	ε_{local}	$T_{B,coupled}$ (s)	$T_{1,uncoupled}$ (s)	$D_{coupled}$ (µm^2.s^{-1})
6 nm	161	9.9	25.0	2.15×10^{-6}
50 nm	78	4.1	25.0	1.0×10^{-3}
250 nm	80	8.5	25.0	1.0×10^{-3}
500 nm	50	105	25.0	1.0×10^{-3}

b

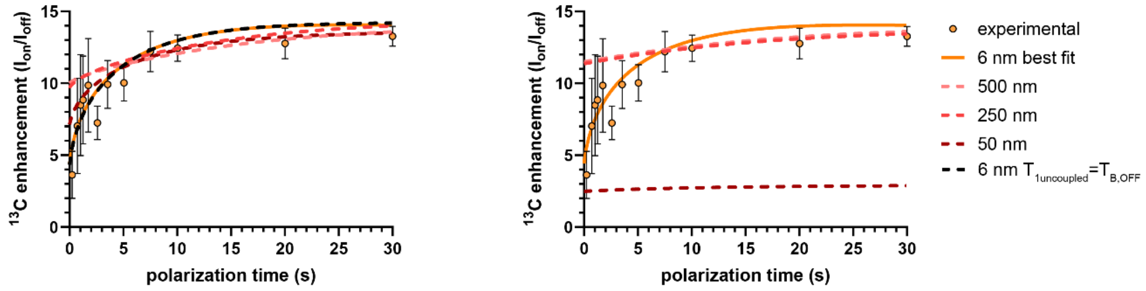

$L_{uncoupled}$	ε_{local}	$T_{B,coupled}$ (s)	$T_{1,uncoupled}$ (s)	$D_{coupled}$ (µm^2.s^{-1})
6 nm	161	9.9	25.0	2.15×10^{-6}
6 nm	180	5.9	8.3	2.15×10^{-6}
50 nm	190	7.1	8.3	2.15×10^{-6}
250 nm	90	10.2	8.3	2.15×10^{-6}
500 nm	55	11.1	8.3	2.15×10^{-6}

$L_{uncoupled}$	ε_{local}	$T_{B,coupled}$ (s)	$T_{1,uncoupled}$ (s)	$D_{coupled}$ (µm^2.s^{-1})
6 nm	161	9.9	25.0	2.15×10^{-6}
50 nm	78	4.1	25.0	1.0×10^{-3}
250 nm	80	8.5	25.0	1.0×10^{-3}
500 nm	50	105	25.0	1.0×10^{-3}

SI-11: *Influence of $T_{1,uncoupled}$ and length of the uncoupled region on the enhancement buildup fitting parameters*. The buildup curves were simulated using a sphere of 5 µm in radius and a base spin diffusion coefficient of 0.001 µm^2.s^{-1}. a) shows simulations where $T_{1,uncoupled}$ is equal to the measured value for undoped cells of 25 s. b) shows simulations where $T_{1,uncoupled}$ is equal to the measured $T_{B,OFF}$ value of 8.3 s. All fitting is done with data derived from the buildups of d_6-DMSO at 0.19 nmol/million cellular loading.

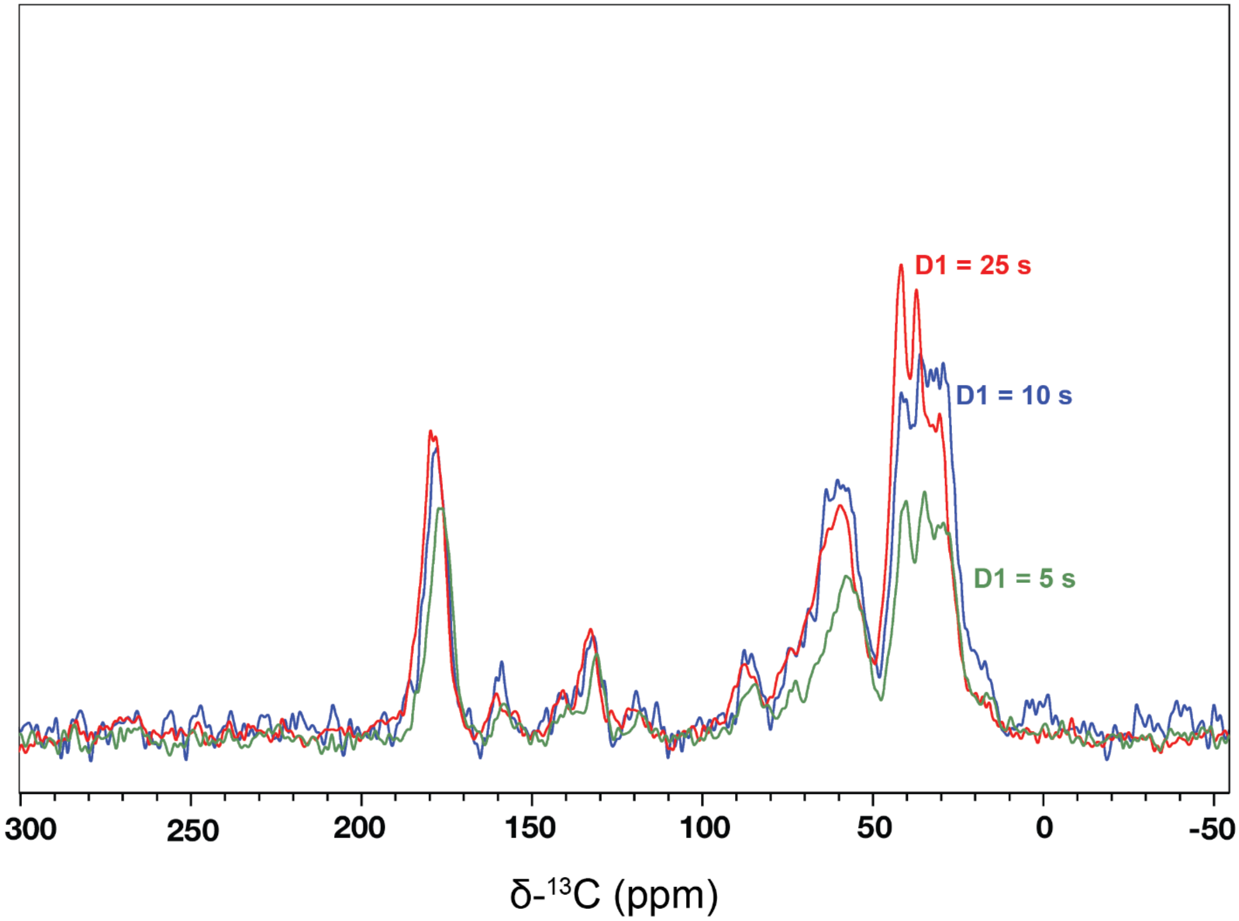

SI-12: *Recycle delay optimization*. ^{13}C spectra of JLat 9.2 T cells loaded with 0.48 nmol/million AsymPol-Chol-AF647 acquired with variable recycle delays. Data was acquired at 400 MHz ^{1}H Larmor frequency with 9 kHz MAS.

References

(1) Pereira de Freitas, R.; Iehl, J.; Delavaux-Nicot, B.; Nierengarten, J.-F. Synthesis of Fullerene Building Blocks Bearing Alkyne or Azide Groups and Their Subsequent Functionalization by the Copper Mediated Huisgen 1,3-Dipolar Cycloaddition. *Tetrahedron* **2008**, *64* (50), 11409–11419. https://doi.org/10.1016/j.tet.2008.09.047.

(2) Aly, M. R. E. S.; Saad, H. A.; Mohamed, M. A. M. Click Reaction Based Synthesis, Antimicrobial, and Cytotoxic Activities of New 1,2,3-Triazoles. *Bioorg. Med. Chem. Lett.* **2015**, *25* (14), 2824–2830. https://doi.org/10.1016/j.bmcl.2015.04.096.

(3) Dasgupta, R.; Steinmetzger, C.; Wilson, A. T.; Chatterjee, S.; Reginsson, G. W.; Sigurdsson, S. Th.; Petzold, K. Targeted NMR Signal Enhancement of RNA by Site-Directed Bis-Nitroxide Labeling. *Proceedings of the National Academy of Sciences* **2026**, *123* (12). https://doi.org/10.1073/pnas.2531087123.

(4) Pinon, A. C.; Schlagnitweit, J.; Berruyer, P.; Rossini, A. J.; Lelli, M.; Socie, E.; Tang, M.; Pham, T.; Lesage, A.; Schantz, S.; Emsley, L. Measuring Nano- to Microstructures from Relayed Dynamic Nuclear Polarization NMR. *The Journal of Physical Chemistry C* **2017**, *121* (29), 15993–16005. https://doi.org/10.1021/acs.jpcc.7b04438.

(5) Maciejewski, M. W.; Schuyler, A. D.; Gryk, M. R.; Moraru, I. I.; Romero, P. R.; Ulrich, E. L.; Eghbalnia, H. R.; Livny, M.; Delaglio, F.; Hoch, J. C. NMRbox: A Resource for Biomolecular NMR Computation. *Biophys. J.* **2017**, *112* (8), 1529–1534. https://doi.org/https://doi.org/10.1016/j.bpj.2017.03.011.
